# Improving Wheat Yield Prediction Using Secondary Traits and High-Density Phenotyping Under Heat-Stressed Environments

**DOI:** 10.3389/fpls.2021.633651

**Published:** 2021-09-27

**Authors:** Mohammad Mokhlesur Rahman, Jared Crain, Atena Haghighattalab, Ravi P. Singh, Jesse Poland

**Affiliations:** ^1^Department of Plant Pathology, Throckmorton Plant Sciences Center, Kansas State University, Manhattan, KS, United States; ^2^Stakman-Borlaug Center for Sustainable Plant Health, University of Minnesota, St Paul, MN, United States; ^3^International Maize and Wheat Improvement Center (CIMMYT), Texcoco, Mexico; ^4^Department of Plant Pathology, Wheat Genetics Resource Center, Throckmorton Plant Sciences Center, Kansas State University, Manhattan, KS, United States

**Keywords:** canopy temperature, grain yield prediction, heat-stress, high-throughput phenotyping, normalized difference vegetation index, wheat

## Abstract

A primary selection target for wheat (*Triticum aestivum*) improvement is grain yield. However, the selection for yield is limited by the extent of field trials, fluctuating environments, and the time needed to obtain multiyear assessments. Secondary traits such as spectral reflectance and canopy temperature (CT), which can be rapidly measured many times throughout the growing season, are frequently correlated with grain yield and could be used for indirect selection in large populations particularly in earlier generations in the breeding cycle prior to replicated yield testing. While proximal sensing data collection is increasingly implemented with high-throughput platforms that provide powerful and affordable information, efficient and effective use of these data is challenging. The objective of this study was to monitor wheat growth and predict grain yield in wheat breeding trials using high-density proximal sensing measurements under extreme terminal heat stress that is common in Bangladesh. Over five growing seasons, we analyzed normalized difference vegetation index (NDVI) and CT measurements collected in elite breeding lines from the International Maize and Wheat Improvement Center at the Regional Agricultural Research Station, Jamalpur, Bangladesh. We explored several variable reduction and regularization techniques followed by using the combined secondary traits to predict grain yield. Across years, grain yield heritability ranged from 0.30 to 0.72, with variable secondary trait heritability (0.0–0.6), while the correlation between grain yield and secondary traits ranged from −0.5 to 0.5. The prediction accuracy was calculated by a cross-fold validation approach as the correlation between observed and predicted grain yield using univariate and multivariate models. We found that the multivariate models resulted in higher prediction accuracies for grain yield than the univariate models. Stepwise regression performed equal to, or better than, other models in predicting grain yield. When incorporating all secondary traits into the models, we obtained high prediction accuracies (0.58–0.68) across the five growing seasons. Our results show that the optimized phenotypic prediction models can leverage secondary traits to deliver accurate predictions of wheat grain yield, allowing breeding programs to make more robust and rapid selections.

## Introduction

Wheat accounts for 26% of world cereal production and 44% of total cereal consumption (McGuire, 2015). Rapid economic and income growth, urbanization, and globalization are leading to dramatic dietary shifts, especially in Asia as consumers are increasing their consumption of wheat products (Pingali, 2007). Wheat production needs to increase to meet the combined growing population and expanding demand by the middle of this century (Tilman et al., 2011). Currently, wheat yield gains are estimated to be 0.9% per year, much less than the 1.5% per year, which is required to meet the projected 60% increase in global production needed by 2050 (Reserach Program on Wheat, 2016). At the current rate, the global production of wheat may only increase by 38%, which is far short of the projected demand. Additionally, the effect of climate change, such as less favorable growing conditions, may even further reduce wheat production (Gammans et al., 2017). Up to 6% yield declines are projected in wheat for each degree Celsius temperature increase if adaptive measures such as improved germplasm are not realized (Zhao et al., 2017).

While wheat is globally distributed and faces a variety of biotic and abiotic challenges, in South Asia, heat is the most important stress and critical yield limitation. Terminal heat stress is also a common problem in temperate regions where 40% of the world's wheat is produced. In these areas, the temperature that ranges from 32 to 38°C can cause up to a 50% grain yield reduction (Asseng et al., 2011). Heat stress is a regulated physiological process that can affect a range of plant phenotypes such as canopy temperature (CT) (Ayeneh et al., 2002). Fundamental research has shown that this response is highly complex and differs at the tissue (Thomason et al., 2018), species (Kotak et al., 2007), and developmental stage (Tricker et al., 2018), suggesting that heat tolerance is a physiologically and genetically complex trait.

Temperatures above the optimum level are deleterious and cause irreversible damage, with the duration and magnitude of temperature exposure determining the severity of yield loss. In controlled studies with supraoptimal temperatures, a 3–5% yield loss for every 1°C increase of mean temperature above 15°C has been observed (Gibson and Paulsen, 1999). In addition to reducing grain yield, high temperatures can reduce individual grain mass by up to 23% (Stone and Nicolas, 1994), further impairing grain yield and quality (Teixeira et al., 2013). Many of the global wheat production areas already have supraoptimal temperature conditions, and global temperatures are predicted to further increase between 1.7 and 4.8°C by the end of the century (Pachauri et al., 2014). Thus, increasing grain yield under heat stress is a major global objective, and more efficient breeding methods and technology are needed to increase the rate of genetic gain in heat-stressed environments.

The complexity of heat stress means that the breeding programs cannot use a single strategy to improve heat tolerance. Some plant adaption mechanisms to avoid and minimize heat stress include early flowering (Ishimaru et al., 2010) and stomatal closure (Liu et al., 2018). The difference in the expression of these traits provides an opportunity to improve wheat if this beneficial genetic variation can be accurately measured. Traditionally, before the discovery of DNA and molecular markers, plant breeders selected promising lines only on the basis of phenotype. By generating large numbers of crosses and evaluating successive generations in a wide range of environments, superior individuals could be identified. While great improvements have been made in this fashion, as the number of lines to evaluate increases, breeders are faced with the challenge of precisely phenotyping large populations within a short time to identify the best progeny.

With the advent of low-cost, high-throughput genotyping technologies, breeders have access to high-density genomic data (Morrell et al., 2012). While molecular markers have aided in breeding objectives (Bernardo, 2008), breeding programs continue to face a combined challenge of characterizing breeding lines precisely and rapidly (McMullen et al., 2009; Araus and Cairns, 2014). Unraveling complex traits, such as heat stress, requires precise, and accurate phenotypic data to connect the phenotype to the genotypic data (Cobb et al., 2013). Phenotyping is now considered the bottleneck of crop improvement, but it is crucial to fully realize the benefits of plant breeding (Araus and Cairns, 2014).

Increasing grain yield, especially under extreme terminal heat stress, is a primary goal of the national breeding program in Bangladesh. While grain yield is the primary trait of interest, it can be estimated using remote or proximal sensing data (Lillesand et al., 2014). Any trait that is correlated with the primary trait can be considered a secondary trait in selection and can potentially be used to reduce evaluation time and cost (Rutkoski et al., 2016). If the secondary traits can be accurately phenotyped within the breeding program, these secondary traits can be used to predict the primary trait and to improve genetic gain particularly earlier in the breeding cycle before advancement to replicated yield trials. Two potential secondary traits that are amendable to high-throughput measurements include spectral reflectance and canopy temperature (CT) (Pask et al., 2012).

Remote sensing of spectral reflectance is based on the ability to measure the electromagnetic reflectance of plants. The cells and tissues of plants have wavelength-specific absorbance and reflectance properties that make spectral reflectance a trait that can be rapidly and quantitatively measured (Montesinos-López et al., 2017). Remote sensing has been widely used in agriculture with different vegetation indices providing a non-destructive, real-time measure of crop growth. The normalized difference vegetation index (NDVI) is one of the most commonly used vegetation indices based on the reflectance of red and near-infrared light. It can be used to characterize crop growth stages, evaluate crop density, and predict crop yield (Rutkoski et al., 2016). In crops, such as maize, wheat, sorghum, and barley, scientists have identified significant correlations between biomass and NDVI with some correlation coefficients above 0.70 (Chen et al., 2011). The values of NDVI, especially 2–3 weeks before and after heading, are highly correlated with grain yield in wheat (Babar et al., 2006).

Another trait that can be used to evaluate crop status is CT. Crop CT is the surface temperature of the plant canopy and is related to the amount of transpiration that results in evaporative cooling. CT plays an important role in the observation of the crop-water relationship, which is a factor of crop yield, and CT has been shown to have the potential for selecting heat- and drought-tolerant genotypes in stressed environments (Reynolds et al., 2009). Several important biological factors such as root length and biomass, stomatal conductance, number of stomata, metabolic activities, and photosynthate translocation result in variation in CT between different genotypes (Reynolds et al., 2012). Mason et al. (2013) suggested that CT is a complex trait controlled by loci of small effect with most of the loci having pleiotropic effects on traits such as plant height (PH) and days to heading (DTHD). Even though the exact mechanism of CT difference is unresolved, research has shown that the correlation between CT and grain yield in wheat is generally negative under heat-stressed environments providing selection strategies to identify heat-tolerant lines (Amani et al., 1996; Gutierrez et al., 2010; Mason and Singh, 2014).

While CT can be easily measured using handheld infrared radiometers (Pask et al., 2012) and often has moderate heritability (Lopes et al., 2012), the application of CT in breeding has been limited due to the inconsistent nature of the CT measurements. CT is impacted by a variety of environmental factors such as solar radiation intensity, atmospheric temperature, humidity, soil moisture, and wind speed, which can quickly change throughout the day (Reynolds et al., 2012). The complexities of CT measurements suggest that it is important to determine how to effectively use CT to select better yielding lines in large wheat breeding programs under heat-stressed environments.

Both CT and NDVI can be measured multiple times throughout the growing season that gives a powerful approach to capture the temporal dynamics of the growing crop. Using just a single measurement to evaluate lines in a breeding program neglects the temporal dynamics of plant growth and development (Crain et al., 2018). Incorporating a combination of multiple variables that show a strong correlation between secondary and primary traits can be used to develop precise inferences about crop phenotypes such as grain yield prediction using secondary traits (Guo et al., 2014). While NDVI and CT have been advocated for plant selection, minimal work has been carried out on incorporating multiple measurements into selection decisions.

As precision phenotyping becomes more routine in breeding programs, new challenges include how to best utilize and translate these data into improved prediction models and selection strategies (Tester and Langridge, 2010). The objective of our study was to evaluate how dense, temporal phenotypic measurements from the proximal sensing of NDVI and CT as well as other agronomic traits could be used within the national plant breeding programs of Bangladesh to assess line performance in heat-stressed environments. Additionally, an emphasis was placed on statistical modeling that could account for highly correlated measurements of secondary traits.

## Materials and Methods

### Experimental Design and Field Management

We evaluated different sets of 540 advanced lines from the International Maize and Wheat Improvement Center (CIMMYT) in each of the five growing seasons (i.e., 2015–16, 2016–17, 2017–18, 2018–19, and 2019–20) in Bangladesh. Each year, the sets of 540 lines from CIMMYT were evaluated as new heat-tolerant material became available, and additionally, there were seven different local checks including BARI Gom 26 or BARI Gom 30, which served as the benchmark check variety of Bangladesh. All lines were evaluated in the high heat-stressed environment at the Regional Agricultural Research Station (RARS), Bangladesh Agricultural Research Institute (BARI), Jamalpur, Bangladesh (N 24.93, E 89.93, 23 masl). The climate of this region is hot and humid leading to an overall heat-stressed environment, classified as ME5A according to the CIMMYT wheat mega-environment classification system (Rajaram et al., 1993).

To manage spatial variability, the lines were placed in multiple trials each growing season. Each trial consisted of 60 entries including 53 breeding lines and 7 check varieties. Complete trials were planted within a given day each year with planting dates for each season of December 4–8, 2015; November 25–28, 2016; November 29–30, 2017; November 28, 2018; and December 05, 2019. The trials were arranged in an alpha lattice design with two replications for a total of 120 plots in each trial. Each replication was composed of 12 blocks with 5 entries randomly assigned to each block. The plots were composed of 6 rows of 4.17-m length and on 20-cm row spacing for a total experimental plot size of 5 m^2^. Plots were separated by a 40-cm alley. The 2015–16 season had a total of 10 trials. Subsequent years had a total of 11 trials, with the 11th trial representing the second-year testing of the highest performing lines from the previous season.

The recommended agronomic practices of the Bangladesh Wheat Research Center were followed during the growing season. Fertilizer application consisted of 100:26:50:20:5:1 kg/ha of N:P:K:S:Zn:B, respectively, each year. Irrigation was applied as needed to prevent water deficit. In the 2015–16 growing season, three irrigations were applied at tillering, heading, and grain filling, while from 2016–17 to 2019–20, two irrigations were applied at tillering and booting (Zadoks et al., 1974). Manual weeding was completed every season to keep the plots weed-free. No pesticides were applied during the growing seasons.

### Trait Measurement

We considered grain yield as the primary trait, CT and NDVI as sensor-based secondary traits, and all other traits as agronomic traits. The total grain yield of each of the plots was harvested, dried, weighed, and divided by the plot size (5 m^2^) to get yield (kg/m^2^) and then converted into metric tons per hectare. Throughout the growing season, phenotypic data were recorded for agronomic traits such as ground coverage (GrndCov), DTHD, days to maturity (DAYSMT), PH, grains per spike (GRNSPK), leaf blight disease due to *Helminthosporium* severity (HELSPSEV), number of spikes per unit area (SN), number of spikelets per spike (SPLN), spike length (SPKLNG), and thousand grain weight (TGW). GrndCov was a visual estimation of ground covered by the biomass of the crop beginning 30 days after sowing and continuing at 15-day intervals. DTHD was recorded as the number of days to when 50% of total plants in a plot had extended a spike from the leaf sheath. DAYSMT was recorded when 80% of the plants in a plot had peduncles that had turned from green to golden. Plant height was measured as the length from ground level to the apex of the spike excluding awns. The total number of grains from five spikes was counted and divided by five to get the number of GRNSPK. The HELSPSEV was scored according to the scale for appraising foliar intensity of wheat diseases (Saari and Prescott, 1975). The number of total heads per square meter (i.e., SN) was assessed by measuring the number of spikes counted from a 3.5-m-long 20-cm spacing (0.7 m^2^) and converted into the number of spikes per square meter. SPKLNG was measured on a representative spike within the plot as the length from the base to the tip of a spike excluding awns.

Secondary traits of CT and NDVI data were collected from 8 to 15 times during the growing seasons (8, 14, 12, 13, and 15 time points for the 2016–17, 2017–18, 2018–19, and 2019–20 seasons, respectively). The measurements represented plant growth from tillering through senescence (Zadoks et al., 1974) with measurements taken from 11 a.m. to 2 p.m. corresponding to solar noon on each day of observation. CT was measured using a handheld infrared thermometer (IRT) (Apogee, Logan, UT, USA), which provided a high accuracy, non-contact surface temperature measurement from −30 to 65°C with a precision of ±0.124°C. The IRT readings were taken at a 30° angle from the horizon for measurement and 70 cm above the crop canopy (Pask et al., 2012). The IRT functions at 0.6 hertz, but only the average CT was recorded for each measurement. NDVI was collected using a GreenSeeker handheld sensor (Trimble Inc. Sunnyvale, CA, USA). The GreenSeeker was used by passing the sensor 75 cm over the crop canopy. Two-person teams were employed for CT and NDVI collection, with one person operating the instrument and the other person recording the data. It took ~3 h with two teams (i.e., four people) to measure CT and NDVI of all plots. The data were recorded in the Field Book program (Rife and Poland, 2014).

### Data Analysis

All analyses were completed in R software (Team, 2017) by using packages including lme4 (Bates et al., 2015), leaps (Lumley, 2017), tidyverse (Wickham et al., 2019), glmnet (Friedman et al., 2010), plyr (Wickham, 2011), ggplot2 (Wickham, 2016), caret (Williams et al., 2018), PerformanceAnalytics (Peterson et al., 2014), and readr (Wickham et al., 2017).

### Statistical Analysis

A mixed model to account for the trial design was used to obtain the best linear unbiased estimators (BLUEs) for each genotype using the following model fit separately for each trial:


(1) $$\begin{array}{l} y_{ij}=\mu +g_{i}+r_{j}+b_{n\left(j\right)}+e_{ij} \end{array}$$

where *y*_*ij*_ is the observed phenotypic response variable (GRYLD, CT, …, NDVI) for the *i*th genotype, *j*th replicate; μ is the overall mean of the individual trial; *g*_*i*_ is the fixed effect of *i*th genotype (line) with *i* taking the values 1–60; *r*_*j*_ is the random effect of *j*th replicate with *j* corresponding to 1 or 2 with a normal distribution *N*(0, $\sigma_{r}^{2}$); *b*_*n*_ is the random effect of *n*th block, nested within replicate *j*, where *n* ranges from 1 to 12 distributed as *N*(0, $\sigma_{n}^{2}$); and *e*_*ij*_ is the residual effect for genotype *i* in replicate *j* with a normal distribution *N*(0, $\sigma_{e}^{2}$). BLUEs were calculated for each site year individually.

To estimate heritability for each trial, a random term for genotype was used in equation (1), resulting in variance components used to calculate broad-sense heritability. The heritability was estimated using the following formula (Holland et al., 2003):


(2) $$\begin{array}{l} {\text{H}}^{2}=\frac{\sigma_{g}^{2}}{\sigma_{g}^{2}+\frac{\sigma_{e}^{2}}{r}} \end{array}$$

where $\sigma_{g}^{2}$ is genotypic variance, $\sigma_{e}^{2}$ is residual model variance, and *r* is the number of replications, which is two. The heritability estimates were calculated for all agronomic traits during the growing season and for each of the time points of NDVI and CT observations. In addition to calculating heritability on a trial basis, we estimated BLUEs and variance components across the full experiment each year for each trait using the following model:


(3) $$\begin{array}{l} y_{ijk}=\mu +t_{k}+g_{i\left(k\right)}+r_{j\left(k\right)}+b_{l\left(ij\right)}+\varepsilon_{ijk} \end{array}$$

where *y*_*ijk*_ is the phenotype of the trait of interest for *i*th genotype, *j*th replicate, and *k*th trial; μ is the overall mean of the population; *t*_*k*_ is the random effect of the trial with *k* taking values 1–11 with a normal distribution *N*(0, $\sigma_{k}^{2}$); *g*_*i*_ is the random effect of *i*th genotype (line) nested within trial with *i* taking the values 1–60 with a normal distribution *N*(0, $\sigma_{i}^{2}$); *r*_*j*_ is the random effect of *j*th replicate nested within trial with *j* corresponding to 1 or 2 with a normal distribution *N*(0, $\sigma_{j}^{2}$); *b*_*l*_ is the random effect of *n*th block, nested within trial *i* and replicate *j*, with *n* from 1 to 12 distributed as *N*(0, $\sigma_{n}^{2}$); and ε_ijk_ is the residual effect for the *i*th genotype *j*th replicate in the *k*th trial with normal distribution *N*(0,$\sigma_{e}^{2}$).

### Statistical Models for Grain Yield Prediction

Using the BLUEs for each trait, four different statistical models were used to predict grain yield using multiple measurements of NDVI, CT, and agronomic traits. The models included stepwise regression and three shrinkage regression models of ridge regression, least absolute shrinkage and selection operator (LASSO) regression, and ElasticNet regression (Hastie et al., 2001). In all models, we used all the secondary traits and agronomic traits collected from the field to predict grain yield. The stepwise regression performed forward selection followed by the backward elimination (Friedman et al., 2010, pp. 58–60). The shrinkage models function by shrinking the estimated effects toward zero. These models add a penalty that allows variables to have a coefficient close to or equal to zero. The tuning parameter lambda thus determines the amount of shrinkage. The LASSO regression model performs L1 regularization (i.e., the absolute value of the residual error term), and it can select variables by eliminating variables with a coefficient of zero (Hastie et al., 2001, p. 68). The ridge regression performs L2 regularization (i.e., the squared value of residual error term), and the coefficients cannot be zero, thus retaining all variables in the model (Friedman et al., 2010, pp. 61–68). The penalty for the ElasticNet regression is a combination of ridge and LASSO regression, allowing for both variable shrinkage and feature selection (Hastie et al., 2001, pp. 72–73; James et al., 2013). The models were built in an iterative process; for each year, we evaluated models with NDVI only, CT only, and all secondary and agronomic traits together.

For each model, a cross-validation approach was evaluated to determine the predictive ability for yield using the trial structure of the CIMMYT trials. As related lines (e.g., full sibs) are evaluated in the same trial, this approach prevents highly related, full- or half-sibling lines, from predicting their own performance. In the cross-validation scheme, all entries from 10 (9 in 2015–16 and 2018–19 seasons) trials were used to fit the model, and the prediction was completed on the 11th (10th in 2015–16 and 2018–19 seasons) trial. This process was repeated by dropping a single trial fitting the model and predicting the left-out trial until all entries had been predicted. The reported prediction accuracy was assessed as the correlation between the predicted value and the BLUEs for grain yield.

## Data Availability Statement

All phenotypic data and code for analysis have been placed in the Dryad Digital Repository available at: https://doi.org/10.5061/dryad.vdncjsxrz.

## Results

Over five seasons where we evaluated ~2,700 lines along with a local check variety for grain yield, which ranged from a low of 2.4 to a high of 3.5 ton ha^−1^. Overall, the 2020 field season had the highest average yield whereas 2016 was the lowest yield (Supplementary Figure S1). In general, these yields are lower than experienced in most global areas where the mean global wheat yield is estimated to be 3.4 ton ha^−1^ (Ritchie and Roser, 2013). This is likely due to the high heat stress found in the Bangladesh environments. To identify new candidate varieties for farmers, we evaluated the CIMMYT germplasm compared to the local check varieties. Within the CIMMYT germplasm, each year there were lines that exceeded the local check, with some lines being highly superior. For each season of the 540 lines evaluated, 24% to 56% of the lines were higher yielding than the check varieties (Supplementary Figure S2). Based on these tests and observations, there are opportunities to improve wheat yield in Bangladesh and heat-stressed areas.

### Broad-Sense Heritability

We observed moderate-to-high broad-sense heritability (repeatability) for grain yield and other agronomic traits, across the five seasons from 2015–16 to 2019–20 when considering the entire experiment (all trials together) (Table 1) and also on an individual trial basis (Supplementary Tables S1–S5). For the agronomic traits such as DTHD, DAYSMT, and PH, we observed a consistent and high heritability. The highest heritability was recorded from DTHD (*H*^2^ = 0.97; followed by DAYSMT, *H*^2^ = 0.90) across the trials and growing seasons.

**Table 1:** Broad-sense heritability of agronomic traits and correlation between agronomic traits and grain yield (GRYLD) for five growing seasons from 2015–16 to 2019–20 for wheat grown in Bangladesh.

**Traits**	**2015–16**		**2016–17**		**2017–18**		**2018–19**		**2019–20**	
	***H* ^**2**^**	***r***	***H* ^**2**^**	***r***	***H* ^**2**^**	***r***	***H* ^**2**^**	***r***	***H* ^**2**^**	***r***
Days to heading	0.94	−0.05 *ns*	0.94	−0.29^***^	0.97	−0.32^***^	0.94	−0.16^***^	0.96	−0.19^***^
Days to maturity	0.72	0.30^***^	0.90	−0.01 *ns*	0.90	−0.01 *ns*	0.88	−0.04 *ns*	0.87	0.08^*^
Plant height	0.51	0.35^***^	0.57	0.24^***^	0.35	0.27^***^	0.28	0.31^***^	0.44	0.38^***^
Number of spikes per m^2^	0.75	0.33^***^	0.03	0.22^***^	0.35	0.41^***^	0.05	0.06 *ns*	0.18	0.36^***^
Number of spikelets	0.31	0.10^*^	0.28	0.04*ns*	0.19	−0.03 *ns*	0.29	−0.10^*^	0.15	0.00 *ns*
Kernels per spike	0.80	0.14^***^	0.36	0.16^***^	0.19	−0.01 *ns*	0.07	0.14^***^	0.15	0.10^*^
Thousand kernel weight	0.47	0.25^***^	0.55	0.28^***^	0.61	0.49^***^	0.52	0.17^***^	0.41	0.33^***^
Grain yield	0.72	−	0.66	−	0.56	−	0.30	−	0.39	−
Spike length	0.42	0.05 *ns*	0.36	0.05 *ns*	0.30	0.20^***^	0.29	0.00 *ns*	−	−

*H^2^= Broad-sense heritability, and r = Correlation between grain yield and agronomic trait. ns not significant*.

* *Significant at the 0.05 probability level*.

** *Significant at the 0.01 probability level*.

*** *Significant at the < 0.001 probability level*.

**Figure 1:**
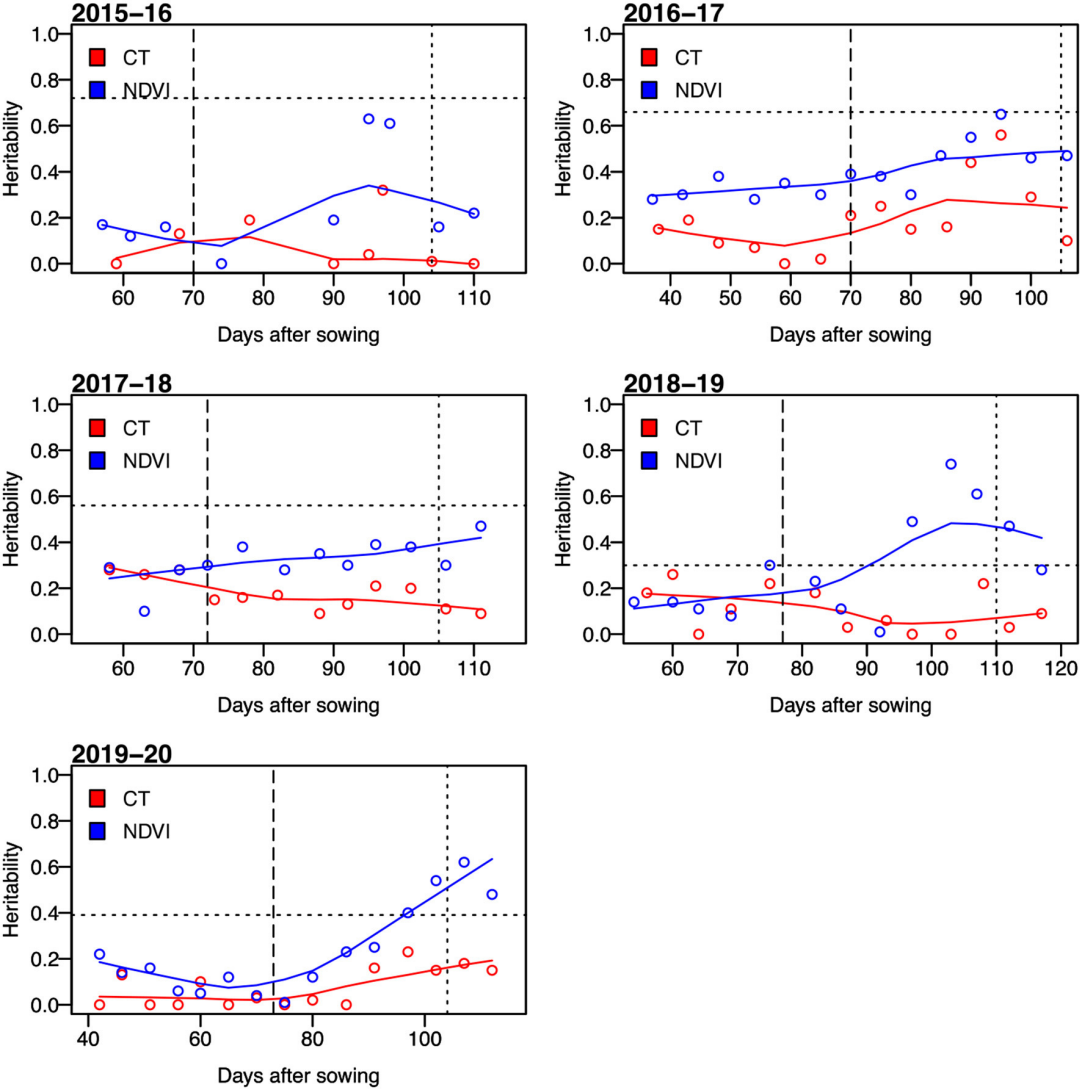
Broad-sense heritability of the normalized difference vegetation index (NDVI) and canopy temperature (CT) for days after sowing in five growing seasons from 2015–16 to 2019–20. The horizontal dotted lines represent the heritability of grain yield. The vertical dashed lines indicate average days to heading, and the dotted lines represent the average days to physiological maturity.

**Figure 2:**
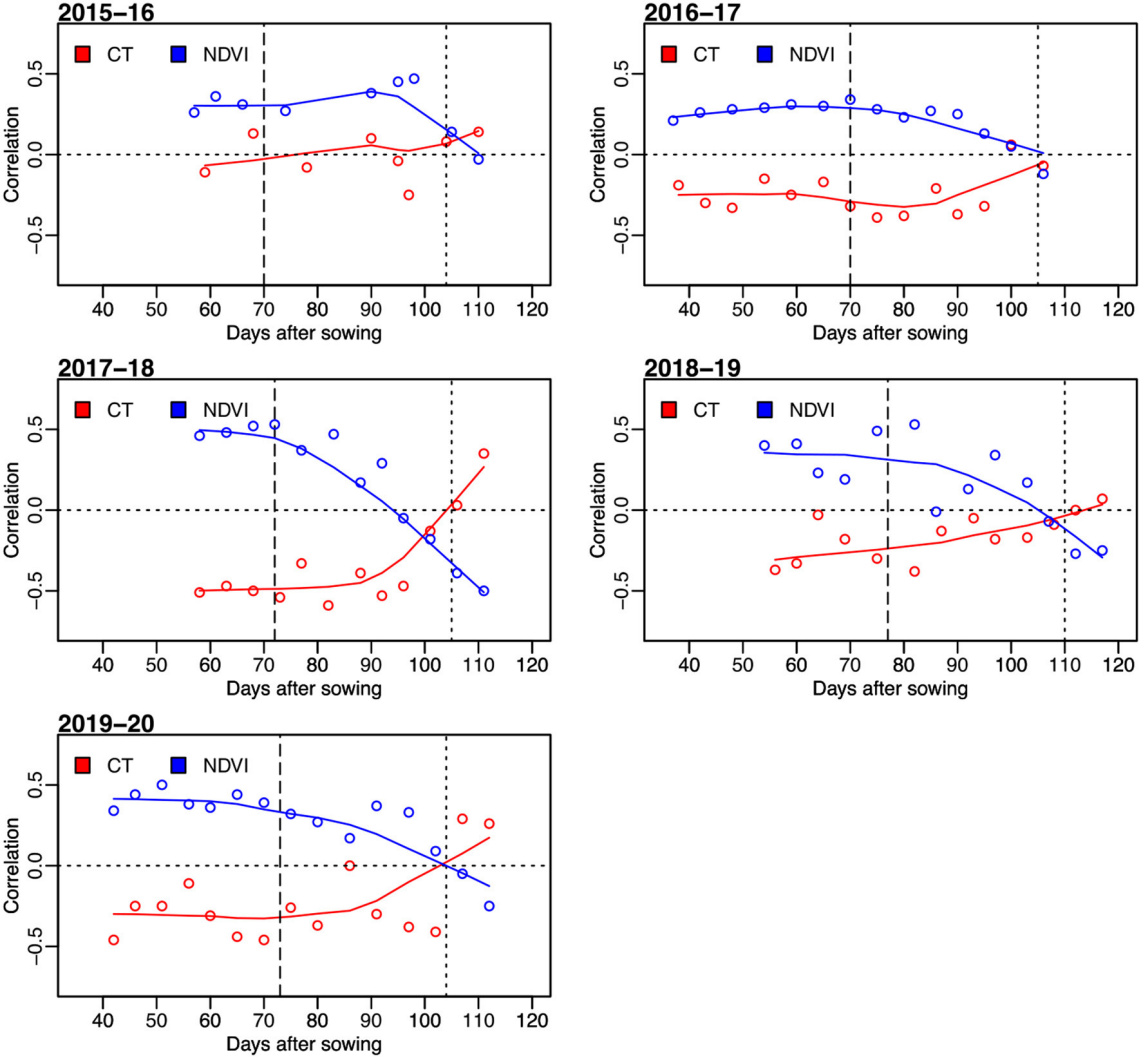
Correlation between grain yield and sensor-based secondary traits of NDVI and CT for observations on days after sowing in five wheat growing seasons from 2015–16 to 2019–20. The horizontal dotted lines represent the correlation value of 0. The vertical dashed lines indicate average days to heading, and the vertical dotted lines represent the average days to physiological maturity.

For secondary trait measurements, the sensor-based NDVI and CT had heritability ranging from low to high (i.e., from 0 to 0.74). The CT showed a narrower range of heritability compared to that of the heritability of NDVI (Figure 1), but the heritability of CT was almost always lower than that of NDVI. The highest value of heritability was calculated as 0.56 for CT and that for NDVI was 0.74. We observed that the values of heritability for both NDVI and CT were higher at the grain filling stage (i.e., mid-February–mid-March, indicated as two vertical lines on Figures 1, 2) than the early growth stages.

### Correlations Between the Measured Traits

The phenotypic correlations were calculated for all measured agronomic traits, considering all trials together to determine the relationship between them and GRYLD (Table 1). We also calculated the correlations between yield and other agronomic traits for individual trials (Supplementary Tables S6–S10). DTHD showed a moderate but negative correlation with grain yield in all the seasons. DAYSMT also showed a negative correlation in three of the five growing seasons. The highest correlation was observed between TGW and GRYLD (*r* = 0.49) followed by GRYLD and SN (*r* = 0.41) in the 2017–18 season. The most consistent correlation of grain yield was observed for PH and TGW across the growing seasons.

The correlation between the measured CTs at individual time points and GRYLD ranged widely with a trend of being strongly negative at the start of the season to a positive correlation at the final measurement (Figure 2). The strongest correlations were recorded from the CT measurement taken during the grain filling stage (i.e., mid-February–mid-March, indicated as two vertical lines on Figures 1, 2). The correlation between CT and GRYLD was more consistent in the 2017–18 season and had the least consistency in the 2015–16 season.

Generally, NDVI tended to show positive correlations with GRYLD at early to middle growth stages (Figure 2). Out of a total of 63 individual days of NDVI measurement at five growing seasons, 58 days showed a significant correlation with GRYLD. The positive correlation, however, changed at the later crop growth stages of all the seasons, where the correlations between NDVI and GRYLD were negative and the correlations between CT and GRYLD were positive.

There were strong correlations between multiple days of secondary trait measurements across seasons, and it was common for the correlation between different time points of NDVI to have correlations of 0.3. Relationships between different CT time points were often not as highly correlated as NDVI.

### Yield Prediction Using Univariate Model

**Figure 3:**
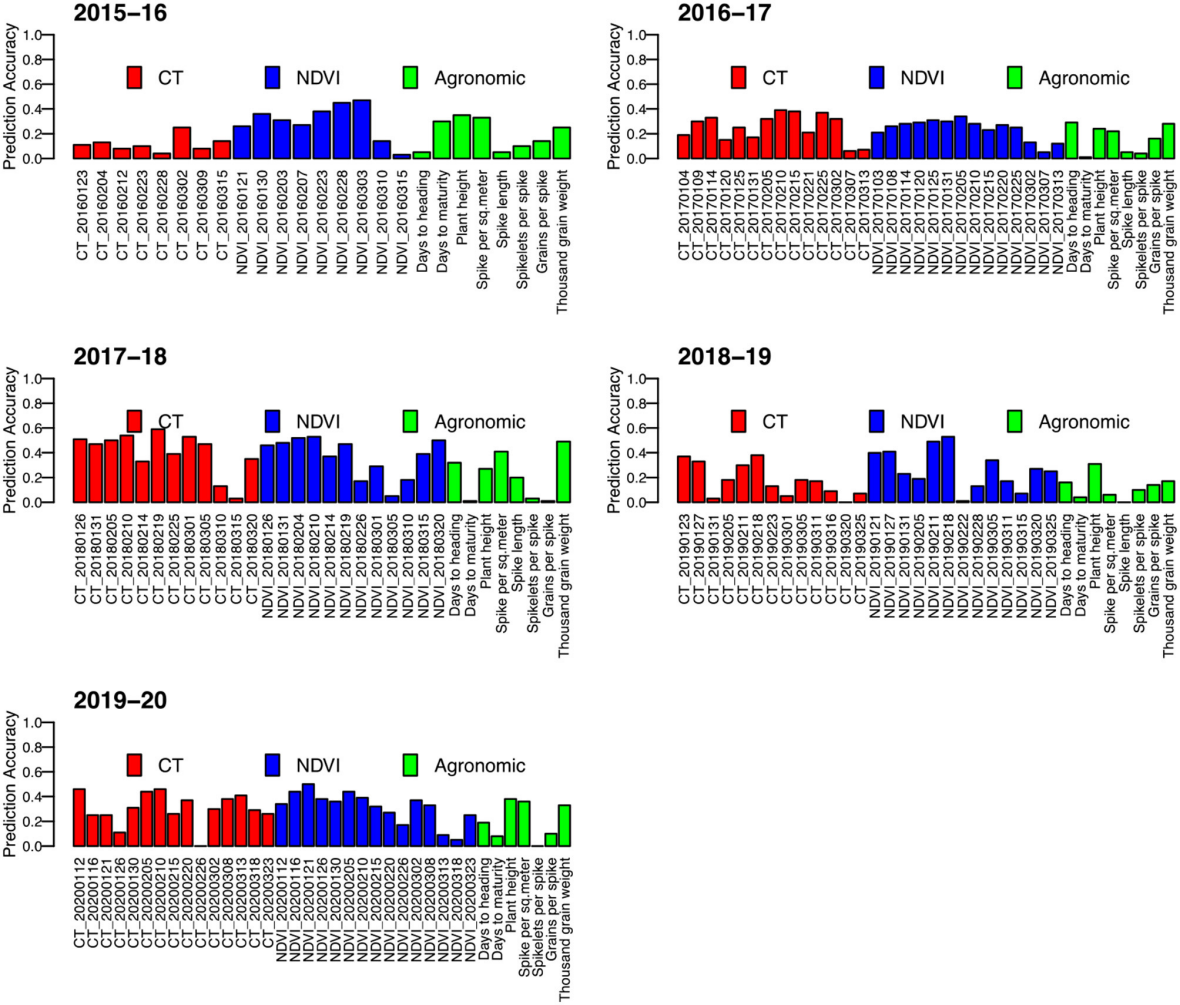
Correlation between predicted grain yield and observed grain yield (prediction accuracy) for five wheat growing seasons in Bangladesh from 2015–16 to 2019–20. Each prediction has been made by using a univariate model with one variable of the phenotypic data.

Yield predictions were developed by implementing a prediction model tested for accuracy with a cross-fold validation strategy. Overall, using a single secondary or agronomic trait, the results were inconsistent with the prediction accuracies ranging from 0 to 0.59. The prediction accuracy of individual secondary traits varied greatly depending on the trait and the time of measurement (Figure 3), with traits measured around grain filling providing the highest values, while traits early or late in the growing season had inconsistent values.

### Yield Prediction Using Multivariate Models

Using four different multivariate models, the accuracy of grain yield prediction was estimated by using a cross-validation strategy where the accuracy was the correlation of the predicted value and the genotypic BLUE. The yield prediction accuracy of the models varied widely from 0.17 to 0.68 (Table 2). When using all traits as predictor variables, it was apparent that the stepwise regression performed similar to shrinkage models, but the proportion of variance explained by the model was always substantially higher than other models. The stepwise regression was consistently the best among the models deployed with LASSO regression, ridge regression, and ElasticNet regression performing similarly.

**Table 2:** Yield prediction accuracies for five wheat growing seasons from 2016 to 2020 in Jamalpur, Bangladesh, using four different multivariate models.

**Yield predictors**	**Models**	**2015–16**		**2016–17**		**2017–18**		**2018–19**		**2019–20**	
		***r***	**R^**2**^**	***r***	**R^**2**^**	***r***	**R^**2**^**	***r***	**R^**2**^**	***r***	**R^**2**^**
Canopy temperature (CT)	Stepwise	0.17	0.07	0.45	0.26	0.34	0.31	0.39	0.14	0.23	0.25
	LASSO	0.17	0.06	0.44	0.24	0.33	0.28	0.39	0.10	0.22	0.23
	Ridge	0.17	0.08	0.44	0.25	0.34	0.30	0.40	0.11	0.23	0.23
	ElasticNet	0.17	0.08	0.44	0.25	0.34	0.30	0.40	0.11	0.23	0.23
Normalized difference vegetation index (NDVI)	Stepwise	0.35	0.20	0.34	0.10	0.43	0.36	0.58	0.36	0.42	0.33
	LASSO	0.35	0.19	0.32	0.08	0.42	0.35	0.58	0.35	0.42	0.32
	Ridge	0.36	0.21	0.32	0.08	0.42	0.35	0.58	0.35	0.41	0.32
	ElasticNet	0.36	0.21	0.32	0.08	0.42	0.35	0.58	0.35	0.42	0.33
NDVI and CT	Stepwise	0.38	0.29	0.48	0.27	0.48	0.42	0.58	0.37	0.43	0.37
	LASSO	0.37	0.27	0.45	0.23	0.44	0.37	0.56	0.31	0.41	0.33
	Ridge	0.38	0.28	0.47	0.25	0.44	0.38	0.56	0.32	0.42	0.33
	ElasticNet	0.38	0.28	0.46	0.24	0.44	0.38	0.57	0.34	0.42	0.33
Agronomic	Stepwise	0.53	0.30	0.53	0.33	0.57	0.48	0.40	0.14	0.60	0.48
	LASSO	0.53	0.29	0.53	0.32	0.57	0.47	0.41	0.13	0.59	0.46
	Ridge	0.53	0.30	0.53	0.32	0.57	0.48	0.41	0.13	0.60	0.47
	ElasticNet	0.53	0.30	0.53	0.32	0.57	0.48	0.41	0.13	0.60	0.47
All Traits	Stepwise	0.58	0.46	0.68	0.50	0.64	0.58	0.65	0.43	0.64	0.57
	LASSO	0.58	0.44	0.66	0.48	0.62	0.52	0.62	0.39	0.62	0.51
	Ridge	0.58	0.44	0.67	0.49	0.62	0.52	0.62	0.39	0.61	0.51
	ElasticNet	0.58	0.43	0.67	0.49	0.62	0.52	0.63	0.39	0.61	0.51

*Yield predictions were based on the cross-fold validation where accuracy is the correlation (r) between the best linear unbiased estimator and the cross-fold validation predicted yield for each trial. R^2^ is the percent variance explained by the prediction model*.

*Agronomic traits include days to heading, days to maturity, plant height, spikes per square meter, spike length, spikelets per spike, grains per spike, and thousand grain weight*.

#### Difference in the Sensor-Based Secondary Trait Selection

Grain yield prediction models were developed iteratively with two distinct secondary traits, namely, NDVI and CT, and other agronomic traits with prediction accuracy in the range of 0.17–0.45 for using CT only (Table 2). Using NDVI, the prediction accuracy was usually higher than using CT alone ranging from 0.32 to 0.58. When we incorporated both NDVI and CT into the model, the prediction accuracy further increased ranging from 0.37 to 0.58. Incorporating all traits together resulted in the highest overall prediction accuracies ranging from 0.43 to 0.68 across the experiment years.

## Discussion

### Phenotypic Evaluation

The national priorities for wheat breeding programs in Bangladesh are focused on improving heat tolerance to develop early maturing varieties with improved yield and superior grain quality. Such breeding efforts necessitate selecting promising lines from large breeding trials. Precise phenotyping is the most important prerequisite to decide which individuals should be selected. The observed heritability for the evaluated physiological high-throughput traits of NDVI and CT was consistent with the previous literature (Reynolds et al., 1994). Most of the CT showed negative correlation, while most days of NDVI observations showed positive correlation and as such should be the useful parameters for selection of superior breeding lines (Babar et al., 2007; Crain et al., 2017). Overall, the sensor-based traits had higher correlations than other agronomic traits and in the context of breeding are amendable to much higher throughput and rapid measurements. However, we also noted that caution should be taken during CT and NDVI data collection as weed population and irrigation management timing could influence the data. Higher weed population could increase NDVI values, and the higher transpiration after irrigation could increase CT expression. Such breeding trial management should be taken into consideration when using these proximal sensing measurements and developing prediction models and selection criteria.

### Modeling Yield Prediction

We evaluated how measured traits could be used to predict grain yield through a variety of statistical models. We used a univariate model to predict grain yield using the phenotypic data as we intended to compare the univariate model to more complex multivariate prediction models. We observed that the univariate models had lower prediction accuracies than any of the multivariate models tested in this study. Using a cross-fold validation, the multivariate stepwise model performed well, with the addition of more variables increasing the power of yield prediction. We found that the stepwise regression was the best among the four multivariate models deployed in predicting grain yield using secondary traits in wheat. The stepwise regression model worked as forward selection and backward elimination processes and finally provides the number of variables that should be included in the regression model. We found that the stepwise regression model excluded some of the secondary traits as they had multicollinearity and were excluded from the model (Supplementary Table S11).

### Application to National Breeding Programs

In a developing country like Bangladesh, genotyping facilities are not yet available. However, field-based phenotyping protocols are available, and these approaches can be implemented across national programs. Hence, within Bangladesh, the phenotypic modeling is directly applicable for the implementation in applied breeding programs for yield prediction and more tractable than selection based only on genomic profiling. Our study supports that large amounts of phenotypic data can be collected with low-cost phenotyping tools.

While the ability to incorporate high-throughput phenotyping (HTP) data in breeding programs is anticipated to increase genetic gains (Haghighattalab et al., 2016; Crain et al., 2018; Krause et al., 2019; Singh et al., 2019; Wang et al., 2020), many of these studies relied on large amounts of resources for both phenotyping and computing. For example, Wang et al. (2019a) used unmanned aerial vehicles to collect HTP imagery. These images were then computationally stitched together followed by the trait extraction using high-performance computers. In the studies by Crain et al. (2018), Rutkoski et al. (2016), and Volpato et al. (2021), expensive phenotyping equipment [i.e., global positioning system (GPS) or multispectral scanners] was used to evaluate plants. To our knowledge, this is the first study that was conducted with low-cost tools and analysis that could be completed with a personal computer (i.e., resources available to many national breeding programs). These methods should be approachable for any breeding program, enabling the data of secondary traits to predict the primary trait of interest and increase selection accuracy. As HTP data collection improves, we anticipate that unmanned aerial vehicle imagery may be able to replace the phenotyping employed in this study. While current results are promising (Krause et al., 2019; Wang et al., 2020), the resources such as skilled technicians, hardware, and software are not at a level that is currently practical in many national breeding programs. While we envision the resources becoming more affordable and user-friendly in the future, the methods we utilized are immediately applicable and eliminate the need to have entire phenotyping research teams that are often suggested for HTP.

In these breeding trials, we evaluated a large diversity of elite breeding germplasm that showed much promise in identifying superior performing candidate varieties for Bangladesh. Overall, there was a high proportion (24–57%) of the evaluated lines that outperformed the local check varieties such as BARI Gom 26 and BARI Gom 30 (Supplementary Figure S2). In addition, the average yield of selected entries (i.e., top 10% of evaluated lines) each year was ≥1 ton above the yield of the benchmark local checks (Supplementary Table S12). These observations and favorable selection results support the upward prospects of continued selection of heat-tolerant breeding materials and the development of new, superior candidate varieties for the supraoptimal temperatures found in Bangladesh. The combined use of more rapid selections with the proposed phenotyping tools and selection methods can further accelerate the identification of these superior candidate varieties.

Our goal was to improve the wheat yield prediction by using secondary traits and statistical models that could accommodate highly correlated variables (Supplementary Table S11). While we investigated models with secondary and agronomic data, the sensor-based data of NDVI and CT can be measured easier than agronomic traits that can require more time and often cannot be measured until the end of the season. Supporting the value of these physiological sensor measurements in breeding, the yield prediction with only the sensor-based data showed prediction power almost as high as the prediction using all traits together. These sensor-based traits are easy to measure repeatedly during the season. This allows breeders to use the sensor-based traits to predict grain yield with flexibility depending on the available equipment and to implement yield prediction on small observation plots. If facilities are limited, NDVI could be used instead of CT for yield prediction. Regardless of the exact type of the sensor-based measurement, breeders will have the ability to increase prediction power by incorporating secondary traits. Breeders can use secondary trait measurements, which are obtained during the growing season, to increase selection accuracies prior to harvesting the plots and ensure that the high-yielding plots are harvested. This is of particular interest if these secondary traits can be measured on smaller plots at earlier generations in the breeding cycle enabling more intense selection prior to lines entering into replicated yield testing (Krause et al., 2020).

## Conclusion

Overall, we found that the proximal sensing of NDVI and CT data was valuable in developing prediction models for yield. When multiple measurements were obtained throughout the growing season, the multivariate prediction models were much more accurate than the models using a single time measurement. Grain yield prediction was also improved by the incorporation of agronomic traits such as DTHD, DAYSMT, and tiller numbers. While less tractable to measure the full suite of agronomic traits (e.g., spikelet number), the incorporation of the routine agronomic measurements into prediction models can be useful for predictions in the breeding program. If future high-throughput technology allows simple image-based measurement of the agronomic traits (Wang et al., 2019a,b), these traits could be measured on large populations and incorporated into prediction models.

This study demonstrated that high prediction accuracy for grain yield can be obtained using the full combination of proximal sensing and agronomic traits with multivariate models. These traits can be measured on small (e.g., <1 m^2^) plots that are used for early generations in the breeding program. Using these same prediction models, it could be possible to generate accurate predictions of grain yield at this stage, where current labor and time constraints prevent harvest assessment. Additionally, using new HTP platforms and unmanned aerial vehicles that can capture NDVI and CT, these measurements can potentially be expanded to tens of thousands of plots. By making predictions and more accurate selections much earlier in the breeding cycle, there is considerable potential to increase genetic gain, particularly for difficult and complex selection targets such as grain yield under heat stress.

## Data Availability Statement

The datasets presented in this study can be found in Dryad Digital Repository at https://doi.org/10.5061/dryad.vdncjsxrz.

## Author Contributions

JP, RS, and MR conceived and designed the study. MR collected and analyzed data. JC analyzed data. AH contributed methods and analysis. RS contributed germplasm. MR, JC, and JP wrote the manuscript. All authors edited and approved the manuscript.

## Funding

This study was based on the support provided by Feed the Future through the U.S. Agency for International Development, under the terms of Contract No. AID-OAA-A-13-00051, by the National Science Foundation under Grant No. 1238187 and 1543958 and the NIFA International Wheat Yield Partnership Grant No. 2017-67007-25933/project accession No. 1011391. MR was supported through the Borlaug Higher Education for Agricultural Research and Development (BHEARD) program.

## Author Disclaimer

Any opinions, findings, and conclusions or recommendations expressed in this study are those of the author(s) and do not necessarily reflect the views of the National Science Foundation, the U.S. Agency for International Development, or the U.S. Department of Agriculture.

## Conflict of Interest

The authors declare that the research was conducted in the absence of any commercial or financial relationships that could be construed as a potential conflict of interest.

## Publisher's Note

All claims expressed in this article are solely those of the authors and do not necessarily represent those of their affiliated organizations, or those of the publisher, the editors and the reviewers. Any product that may be evaluated in this article, or claim that may be made by its manufacturer, is not guaranteed or endorsed by the publisher.

## Abbreviations

BLUE: best linear unbiased estimator
NDVI: normalized difference vegetation index
CT: canopy temperature
DTHD: days to heading
DAYSMT: days to maturity
GRNSPK: grains per spike
GRYLD: grain yield
HELSPSEV: *Helminthosporium* severity
PH: plant height
SN: number of spikes per square meter
SPKLNG: spike length
SPLN: number of spikelets per spike.
