# Supplementary material for: Improving Wheat Yield Prediction Using Secondary Traits and High-Density Phenotyping Under Heat-Stressed Environments
Source: Front Plant Sci. 2021 Sep 27;12:633651. doi: 10.3389/fpls.2021.633651 (PMC8502926; doi:10.3389/fpls.2021.633651)
Supplement: Supplementary file 1 [file Data_Sheet_1.pdf]

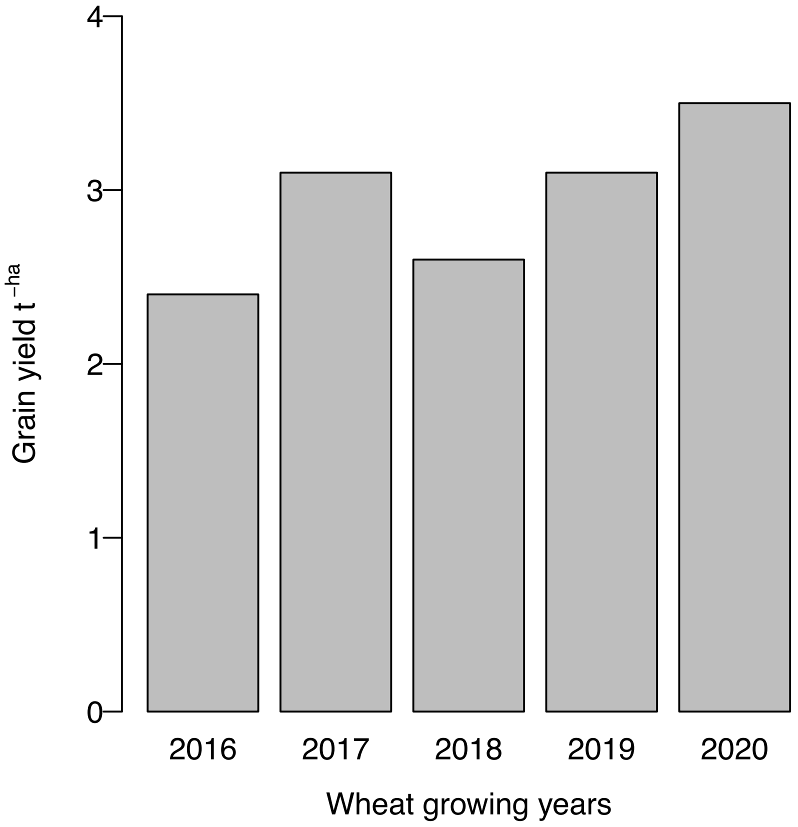

Supplementary Figure 1. Average grain yield of five wheat growing seasons in the experiment field in Bangladesh.

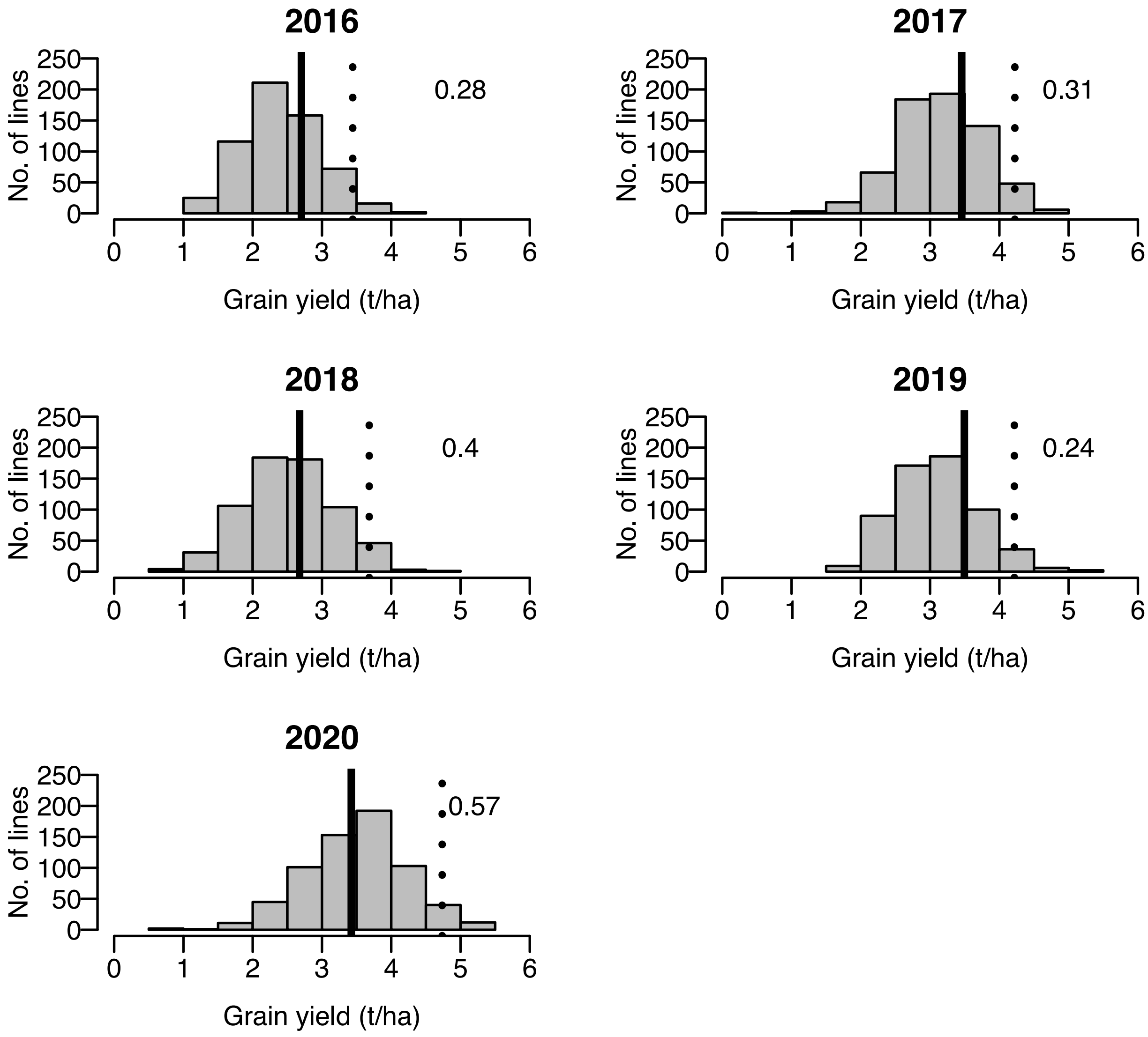

Supplementary Figure 2: A panel plot of the selected lines from genomic prediction trials in five wheat growing season. The solid line is the average grain yield of the national check variety in the trials, which was BARI Gom 26 as local check in 2016 - 2018 and BARI Gom 30 in 2019 and 2020 season. We selected 59 top yielder lines in each year. The dotted line is the average of the selected 59 lines from each year. The text number is the fraction (percentage) of the lines that superseded the yield of the check variety.

Supplementary Table 1: Broad see heritability for the 2015-16 growing season for phenotypic data. Data include normalized difference vegetation index (NDVI) and canopy temperature (CT) measured at multiple times across the growing season (date of measurement) and agronomic traits for wheat grown in multiple yield trials (1-10) in Jamalpur, Bangladesh.

Traits	Trial_1	Trial_2	Trial_3	Trial_4	Trial_5	Trial_6	Trial_7	Trial_8	Trial_9	Trial_10
CT_20160123	0.00	0.00	0.00	0.07	0.00	0.10	0.00	0.00	0.16	0.00
CT_20160204	0.28	0.00	0.00	0.00	0.26	0.39	0.00	0.27	0.04	0.09
CT_20160212	0.00	0.10	0.21	0.33	0.23	0.26	0.27	0.20	0.15	0.00
CT_20160223	0.23	0.09	0.06	0.00	0.00	0.00	0.00	0.03	0.00	0.03
CT_20160228	0.09	0.00	0.20	0.12	0.00	0.43	0.00	0.00	0.00	0.12
CT_20160302	0.53	0.44	0.43	0.00	0.58	0.56	0.28	0.06	0.00	0.50
CT_20160309	0.21	0.00	0.12	0.00	0.21	0.22	0.00	0.17	0.00	0.19
CT_20160315	0.01	0.37	0.01	0.00	0.00	0.03	0.00	0.00	0.31	0.00
NDVI_20160121	0.19	0.00	0.42	0.00	0.00	0.11	0.00	0.33	0.41	0.34
NDVI_20160130	0.21	0.00	0.22	0.06	0.57	0.36	0.00	0.00	0.20	0.27
NDVI_20160203	0.00	0.00	0.21	0.08	0.00	0.48	0.00	0.46	0.00	0.64
NDVI_20160207	0.02	0.06	0.00	0.09	0.38	0.00	0.00	0.00	0.24	0.00
NDVI_20160223	0.49	0.14	0.06	0.29	0.00	0.00	0.40	0.26	0.21	0.00
NDVI_20160228	0.68	0.51	0.68	0.64	0.60	0.71	0.46	0.73	0.44	0.67
NDVI_20160303	0.79	0.60	0.65	0.74	0.76	0.62	0.17	0.64	0.54	0.36
NDVI_20160310	0.00	0.00	0.35	0.00	0.08	0.14	0.42	0.40	0.00	0.26
NDVI_20160315	0.22	0.40	0.00	0.28	0.35	0.30	0.00	0.30	0.12	0.30
Days to Heading	0.98	0.93	0.96	0.91	0.97	0.94	0.96	0.90	0.91	0.91
Days to Maturity	0.80	0.84	0.81	0.66	0.78	0.52	0.50	0.67	0.59	0.55
Plant Height	0.00	0.44	0.64	0.52	0.75	0.50	0.00	0.49	0.63	0.68
Spike Number	0.91	0.83	0.75	0.84	0.74	0.78	0.86	0.64	0.24	0.74
Spike length	0.36	0.38	0.36	0.43	0.42	0.32	0.21	0.68	0.26	0.54
Spikelets per spike	0.47	0.34	0.46	0.27	0.00	0.00	0.51	0.37	0.34	0.31
Grains per spike	0.86	0.71	0.84	0.84	0.82	0.84	0.92	0.74	0.70	0.77
Thousand grain wt.	0.61	0.40	0.42	0.51	0.38	0.62	0.68	0.00	0.38	0.35
Grain yield	0.73	0.58	0.78	0.76	0.65	0.64	0.40	0.73	0.90	0.78

Supplementary Table 2: Broad see heritability for the 2016-17 growing season for phenotypic data. Data include normalized difference vegetation index (NDVI) and canopy temperature (CT) measured at multiple times across the growing season (date of measurement) and agronomic traits for wheat grown in multiple yield trials (1-11) in Jamalpur, Bangladesh.

Traits	Trial_1	Trial_2	Trial_3	Trial_4	Trial_5	Trial_6	Trial_7	Trial_8	Trial_9	Trial_10	Trial_11
CT_20170104	0.19	0.00	0.23	0.00	0.00	0.00	0.33	0.19	0.45	0.26	0.04
CT_20170109	0.10	0.23	0.12	0.38	0.17	0.15	0.16	0.45	0.28	0.01	0.00
CT_20170114	0.00	0.00	0.00	0.01	0.18	0.04	0.00	0.29	0.12	0.11	0.00
CT_20170120	0.07	0.11	0.27	0.42	0.10	0.00	0.33	0.00	0.00	0.31	0.00
CT_20170125	0.00	0.00	0.14	0.10	0.41	0.00	0.06	0.17	0.00	0.00	0.00
CT_20170131	0.00	0.00	0.00	0.00	0.52	0.00	0.00	0.00	0.15	0.00	0.13
CT_20170205	0.52	0.05	0.36	0.40	0.29	0.13	0.04	0.01	0.00	0.26	0.07
CT_20170210	0.05	0.00	0.24	0.17	0.24	0.11	0.23	0.34	0.18	0.56	0.15
CT_20170215	0.42	0.32	0.00	0.00	0.00	0.12	0.25	0.00	0.00	0.40	0.35
CT_20170221	0.31	0.30	0.32	0.42	0.00	0.00	0.53	0.35	0.00	0.24	0.00
CT_20170225	0.54	0.60	0.31	0.49	0.69	0.00	0.63	0.49	0.09	0.37	0.43
CT_20170302	0.61	0.60	0.64	0.73	0.62	0.72	0.69	0.43	0.25	0.55	0.39
CT_20170307	0.14	0.00	0.61	0.55	0.00	0.83	0.77	0.48	0.51	0.56	0.13
CT_20170313	0.00	0.05	0.09	0.19	0.00	0.48	0.00	0.00	0.00	0.00	0.32
NDVI_20170103	0.41	0.12	0.58	0.24	0.48	0.09	0.32	0.39	0.13	0.11	0.18
NDVI_20170108	0.66	0.40	0.63	0.41	0.45	0.08	0.44	0.59	0.00	0.09	0.15
NDVI_20170114	0.52	0.72	0.00	0.51	0.32	0.58	0.46	0.44	0.06	0.30	0.34
NDVI_20170120	0.52	0.56	0.20	0.65	0.18	0.47	0.42	0.21	0.00	0.35	0.28
NDVI_20170125	0.51	0.46	0.34	0.49	0.30	0.55	0.53	0.47	0.20	0.08	0.33
NDVI_20170131	0.36	0.44	0.12	0.53	0.03	0.58	0.29	0.38	0.29	0.00	0.35
NDVI_20170205	0.51	0.51	0.47	0.44	0.06	0.65	0.69	0.38	0.26	0.41	0.27
NDVI_20170210	0.54	0.25	0.53	0.40	0.60	0.52	0.42	0.44	0.00	0.09	0.20
NDVI_20170215	0.58	0.00	0.25	0.07	0.39	0.29	0.22	0.56	0.01	0.35	0.55
NDVI_20170220	0.72	0.03	0.64	0.54	0.65	0.63	0.59	0.49	0.00	0.33	0.30
NDVI_20170225	0.73	0.61	0.42	0.61	0.68	0.65	0.65	0.34	0.06	0.30	0.40
NDVI_20170302	0.77	0.64	0.80	0.50	0.35	0.66	0.78	0.76	0.43	0.74	0.59
NDVI_20170307	0.41	0.50	0.09	0.56	0.40	0.78	0.68	0.23	0.61	0.44	0.25
NDVI_20170313	0.29	0.13	0.43	0.00	0.41	0.79	0.47	0.59	0.63	0.55	0.15
Days to Heading	0.96	0.92	0.95	0.96	0.95	0.96	0.95	0.95	0.88	0.94	0.93
Days to Maturity	0.87	0.86	0.95	0.90	0.95	0.94	0.91	0.89	0.86	0.89	0.84
Plant Height	0.46	0.39	0.06	0.59	0.79	0.57	0.79	0.33	0.38	0.46	0.69
Spike Number	0.09	0.28	0.23	0.21	0.00	0.00	0.00	0.45	0.00	0.00	0.00
Spike length	0.14	0.36	0.03	0.54	0.44	0.29	0.00	0.25	0.38	0.37	0.76
Spikelets per spike	0.14	0.28	0.00	0.28	0.12	0.36	0.48	0.00	0.39	0.60	0.30
Grains per spike	0.53	0.10	0.26	0.32	0.48	0.36	0.44	0.14	0.30	0.44	0.34
Thousand grain wt.	0.69	0.72	0.20	0.70	0.74	0.05	0.75	0.85	0.00	0.53	0.72
Grain yield	0.78	0.71	0.74	0.76	0.75	0.73	0.59	0.70	0.53	0.53	0.20

Supplementary Table 3: Broad see heritability for the 2017-18 growing season for phenotypic data. Data include normalized difference vegetation index (NDVI) and canopy temperature (CT) measured at multiple times across the growing season (date of measurement) and agronomic traits for wheat grown in multiple yield trials (1-11) in Jamalpur, Bangladesh.

Traits	Trial_1	Trial_2	Trial_3	Trial_4	Trial_5	Trial_6	Trial_7	Trial_8	Trial_9	Trial_10	Trial_11
CT_20180126	0.33	0.00	0.00	0.19	0.18	0.37	0.00	0.42	0.00	0.17	0.65
CT_20180131	0.39	0.00	0.22	0.20	0.00	0.55	0.00	0.36	0.05	0.12	0.45
CT_20180205	0.35	0.09	0.00	0.21	0.19	0.39	0.13	0.25	0.00	0.35	0.56
CT_20180210	0.39	0.00	0.00	0.33	0.08	0.32	0.00	0.00	0.00	0.29	0.41
CT_20180214	0.33	0.00	0.15	0.20	0.00	0.29	0.00	0.24	0.17	0.29	0.03
CT_20180219	0.31	0.00	0.13	0.40	0.00	0.26	0.00	0.14	0.00	0.27	0.46
CT_20180225	0.03	0.53	0.00	0.03	0.55	0.17	0.00	0.29	0.20	0.00	0.00
CT_20180301	0.12	0.35	0.00	0.18	0.60	0.14	0.00	0.03	0.17	0.26	0.00
CT_20180305	0.00	0.06	0.00	0.19	0.23	0.14	0.52	0.46	0.10	0.27	0.34
CT_20180310	0.02	0.00	0.13	0.57	0.65	0.32	0.00	0.13	0.20	0.25	0.00
CT_20180315	0.00	0.27	0.12	0.55	0.39	0.00	0.00	0.26	0.00	0.00	0.36
CT_20180320	0.21	0.33	0.29	0.44	0.00	0.17	0.00	0.00	0.00	0.46	0.00
NDVI_20180126	0.41	0.37	0.00	0.26	0.34	0.27	0.26	0.17	0.24	0.30	0.60
NDVI_20180131	0.20	0.27	0.17	0.43	0.16	0.00	0.16	0.00	0.01	0.13	0.70
NDVI_20180204	0.21	0.25	0.30	0.31	0.58	0.09	0.02	0.00	0.33	0.00	0.34
NDVI_20180210	0.41	0.38	0.50	0.58	0.65	0.14	0.32	0.00	0.00	0.32	0.00
NDVI_20180214	0.26	0.59	0.37	0.41	0.49	0.04	0.22	0.26	0.41	0.19	0.16
NDVI_20180219	0.48	0.52	0.26	0.00	0.00	0.49	0.00	0.00	0.10	0.00	0.23
NDVI_20180226	0.13	0.00	0.00	0.05	0.00	0.30	0.35	0.06	0.00	0.00	0.00
NDVI_20180301	0.33	0.68	0.45	0.47	0.78	0.46	0.00	0.16	0.20	0.00	0.14
NDVI_20180305	0.37	0.78	0.27	0.17	0.00	0.74	0.44	0.05	0.05	0.67	0.10
NDVI_20180310	0.43	0.68	0.52	0.72	0.71	0.70	0.66	0.80	0.61	0.71	0.56
NDVI_20180315	0.27	0.72	0.66	0.21	0.57	0.78	0.81	0.80	0.63	0.86	0.63
NDVI_20180320	0.00	0.29	0.14	0.00	0.40	0.44	0.26	0.55	0.00	0.15	0.30
Days to Heading	0.95	0.98	0.93	0.97	0.98	0.97	0.98	0.95	0.96	0.97	0.94
Days to Maturity	0.86	0.96	0.87	0.85	0.94	0.90	0.92	0.84	0.77	0.91	0.86
Plant Height	0.41	0.45	0.17	0.57	0.26	0.30	0.39	0.07	0.52	0.34	0.24
Spike Number	0.41	0.64	0.00	0.12	0.30	0.19	0.11	0.50	0.27	0.35	0.54
Spike length	0.12	0.45	0.44	0.37	0.49	0.26	0.42	0.46	0.00	0.46	0.11
Spikelets per spike	0.20	0.32	0.12	0.34	0.12	0.37	0.00	0.50	0.00	0.37	0.00
Grains per spike	0.00	0.51	0.36	0.41	0.00	0.41	0.00	0.14	0.00	0.10	0.31
Thousand grain wt.	0.39	0.71	0.65	0.54	0.75	0.68	0.64	0.50	0.52	0.70	0.63
Grain yield	0.28	0.63	0.68	0.80	0.71	0.54	0.67	0.08	0.33	0.71	0.66

Supplementary Table 4: Broad see heritability for the 2018-19 growing season for phenotypic data. Data include normalized difference vegetation index (NDVI) and canopy temperature (CT) measured at multiple times across the growing season (date of measurement) and agronomic traits for wheat grown in multiple yield trials (1-10) in Jamalpur, Bangladesh.

Traits	Trial_1	Trial_2	Trial_3	Trial_4	Trial_5	Trial_6	Trial_7	Trial_8	Trial_9	Trial_10
CT_20190123	0.10	0.28	0.11	0.00	0.00	0.22	0.42	0.13	0.22	0.16
CT_20190127	0.12	0.01	0.31	0.29	0.15	0.03	0.34	0.33	0.34	0.16
CT_20190131	0.10	0.34	0.00	0.00	0.00	0.00	0.00	0.00	0.00	0.06
CT_20190205	0.05	0.01	0.00	0.02	0.30	0.00	0.15	0.24	0.26	0.00
CT_20190211	0.00	0.44	0.22	0.28	0.00	0.38	0.28	0.21	0.32	0.05
CT_20190218	0.00	0.29	0.05	0.00	0.34	0.55	0.25	0.30	0.00	0.28
CT_20190223	0.00	0.00	0.00	0.00	0.00	0.20	0.04	0.00	0.00	0.00
CT_20190301	0.18	0.47	0.34	0.00	0.00	0.00	0.00	0.14	0.03	0.11
CT_20190305	0.00	0.00	0.00	0.00	0.00	0.29	0.00	0.00	0.01	0.00
CT_20190311	0.00	0.15	0.33	0.11	0.00	0.22	0.00	0.00	0.00	0.43
CT_20190316	0.00	0.06	0.09	0.00	0.37	0.19	0.34	0.05	0.47	0.48
CT_20190320	0.00	0.02	0.08	0.53	0.28	0.00	0.00	0.00	0.29	0.00
CT_20190325	0.27	0.18	0.00	0.19	0.37	0.36	0.00	0.03	0.00	0.00
NDVI_20190121	0.29	0.30	0.24	0.11	0.24	0.00	0.00	0.02	0.28	0.20
NDVI_20190127	0.29	0.16	0.23	0.08	0.30	0.30	0.29	0.01	0.00	0.22
NDVI_20190131	0.05	0.21	0.10	0.00	0.15	0.00	0.00	0.29	0.22	0.63
NDVI_20190205	0.04	0.12	0.14	0.06	0.00	0.38	0.11	0.04	0.00	0.00
NDVI_20190211	0.00	0.43	0.41	0.00	0.17	0.34	0.45	0.19	0.31	0.39
NDVI_20190218	0.16	0.22	0.46	0.00	0.14	0.05	0.00	0.26	0.27	0.54
NDVI_20190222	0.00	0.12	0.27	0.40	0.11	0.03	0.21	0.22	0.00	0.00
NDVI_20190228	0.44	0.07	0.01	0.00	0.00	0.00	0.00	0.00	0.17	0.15
NDVI_20190305	0.58	0.86	0.45	0.43	0.50	0.05	0.56	0.29	0.35	0.44
NDVI_20190311	0.81	0.82	0.78	0.55	0.75	0.84	0.76	0.69	0.56	0.73
NDVI_20190315	0.73	0.77	0.74	0.69	0.37	0.50	0.40	0.54	0.60	0.69
NDVI_20190320	0.26	0.06	0.44	0.40	0.68	0.49	0.66	0.65	0.01	0.77
NDVI_20190325	0.19	0.11	0.29	0.19	0.66	0.14	0.18	0.20	0.05	0.17
Days to Heading	0.94	0.97	0.94	0.91	0.97	0.94	0.86	0.92	0.90	0.95
Days to Maturity	0.89	0.88	0.93	0.88	0.89	0.87	0.82	0.91	0.85	0.91
Plant Height	0.37	0.11	0.24	0.47	0.61	0.00	0.00	0.32	0.34	0.01
Spike Number	0.06	0.00	0.17	0.00	0.10	0.11	0.28	0.00	0.09	0.16
Spike length	0.00	0.54	0.57	0.49	0.00	0.21	0.07	0.33	0.18	0.00
Spikelets per spike	0.15	0.00	0.42	0.00	0.09	0.41	0.00	0.00	0.00	0.26
Grains per spike	0.00	0.00	0.00	0.00	0.15	0.34	0.07	0.00	0.41	0.41
Thousand grain wt.	0.59	0.55	0.35	0.59	0.17	0.57	0.47	0.48	0.68	0.60
Grain yield	0.24	0.00	0.45	0.01	0.35	0.48	0.61	0.04	0.41	0.36

Supplementary Table 5: Broad see heritability for the 2019-20 growing season for phenotypic data. Data include normalized difference vegetation index (NDVI) and canopy temperature (CT) measured at multiple times across the growing season (date of measurement) and agronomic traits for wheat grown in multiple yield trials (1-11) in Jamalpur, Bangladesh.

Traits	Trial_1	Trial_2	Trial_3	Trial_4	Trial_5	Trial_6	Trial_7	Trial_8	Trial_9	Trial_10	Trial_11
CT_20200112	0.00	0.00	0.31	0.00	0.00	0.00	0.00	0.00	0.17	0.34	0.01
CT_20200116	0.15	0.30	0.14	0.00	0.22	0.00	0.11	0.07	0.00	0.30	0.27
CT_20200121	0.00	0.16	0.19	0.01	0.00	0.00	0.32	0.36	0.03	0.31	0.00
CT_20200126	0.12	0.24	0.10	0.00	0.00	0.20	0.00	0.01	0.11	0.20	0.00
CT_20200130	0.01	0.30	0.27	0.35	0.00	0.00	0.22	0.00	0.00	0.08	0.32
CT_20200205	0.00	0.05	0.30	0.07	0.18	0.00	0.00	0.00	0.00	0.29	0.00
CT_20200210	0.20	0.00	0.44	0.03	0.00	0.00	0.07	0.00	0.00	0.00	0.23
CT_20200215	0.00	0.00	0.43	0.15	0.00	0.00	0.00	0.17	0.00	0.00	0.22
CT_20200220	0.40	0.00	0.26	0.07	0.00	0.07	0.00	0.00	0.12	0.20	0.02
CT_20200226	0.00	0.00	0.00	0.00	0.00	0.00	0.03	0.00	0.00	0.00	0.12
CT_20200302	0.54	0.12	0.11	0.20	0.00	0.00	0.00	0.30	0.00	0.14	0.08
CT_20200308	0.00	0.00	0.42	0.44	0.24	0.40	0.00	0.40	0.02	0.17	0.00
CT_20200313	0.05	0.04	0.28	0.12	0.00	0.37	0.19	0.29	0.37	0.04	0.00
CT_20200318	0.00	0.00	0.05	0.22	0.16	0.27	0.18	0.51	0.14	0.33	0.00
CT_20200323	0.00	0.00	0.30	0.33	0.00	0.45	0.41	0.00	0.00	0.28	0.11
NDVI_20200112	0.07	0.00	0.26	0.07	0.00	0.32	0.00	0.22	0.47	0.35	0.30
NDVI_20200116	0.27	0.24	0.41	0.16	0.00	0.13	0.13	0.06	0.25	0.00	0.26
NDVI_20200121	0.00	0.19	0.28	0.35	0.00	0.07	0.16	0.30	0.20	0.00	0.40
NDVI_20200126	0.12	0.37	0.06	0.00	0.06	0.19	0.14	0.00	0.09	0.00	0.14
NDVI_20200130	0.10	0.18	0.20	0.06	0.18	0.39	0.00	0.00	0.24	0.00	0.00
NDVI_20200205	0.00	0.21	0.00	0.22	0.37	0.13	0.00	0.22	0.24	0.03	0.16
NDVI_20200210	0.23	0.17	0.01	0.00	0.02	0.00	0.00	0.00	0.16	0.27	0.00
NDVI_20200215	0.00	0.05	0.00	0.41	0.12	0.00	0.09	0.06	0.20	0.00	0.00
NDVI_20200220	0.00	0.47	0.00	0.00	0.05	0.22	0.00	0.01	0.36	0.23	0.00
NDVI_20200226	0.10	0.21	0.17	0.00	0.00	0.33	0.00	0.22	0.53	0.48	0.17
NDVI_20200302	0.10	0.30	0.29	0.00	0.09	0.43	0.40	0.56	0.35	0.48	0.07
NDVI_20200308	0.41	0.28	0.46	0.06	0.20	0.47	0.50	0.53	0.64	0.52	0.29
NDVI_20200313	0.59	0.42	0.67	0.62	0.57	0.36	0.69	0.53	0.54	0.65	0.10
NDVI_20200318	0.21	0.70	0.58	0.75	0.64	0.65	0.84	0.82	0.57	0.51	0.18
NDVI_20200323	0.18	0.28	0.39	0.57	0.34	0.38	0.81	0.70	0.33	0.53	0.00
GrndCov_20200112	0.30	0.23	0.17	0.27	0.00	0.32	0.00	0.12	0.00	0.00	0.58

GrndCov_20200206	0.07	0.18	0.15	0.00	0.23	0.00	0.00	0.56	0.33	0.00	0.44
DLA_Feb26	0.45	0.64	0.74	0.71	0.37	0.32	0.49	0.56	0.71	0.47	0.29
DLA_Mar09	0.27	0.40	0.56	0.43	0.69	0.46	0.66	0.55	0.45	0.66	0.69
Days to Heading	0.97	0.94	0.95	0.97	0.95	0.93	0.96	0.96	0.96	0.96	0.95
Days to Maturity	0.88	0.81	0.92	0.95	0.90	0.88	0.86	0.87	0.86	0.83	0.57
Plant Height	0.22	0.35	0.76	0.37	0.47	0.25	0.53	0.52	0.52	0.25	0.47
Spike Number	0.29	0.00	0.01	0.20	0.30	0.62	0.13	0.00	0.40	0.00	0.00
Spikelets per spike	0.20	0.00	0.37	0.02	0.28	0.00	0.45	0.31	0.00	0.22	0.17
Grains per spike	0.23	0.06	0.06	0.28	0.00	0.40	0.01	0.24	0.00	0.23	0.21
Thousand grain wt.	0.48	0.22	0.33	0.58	0.57	0.65	0.37	0.53	0.68	0.00	0.32
Grain yield	0.09	0.18	0.37	0.67	0.09	0.34	0.70	0.51	0.45	0.12	0.17

Supplementary Table 6. Correlation between grain yield and phenotypic traits for the 2015-16 season. Data include normalized difference vegetation index (NDVI) and canopy temperature (CT) measured at multiple times across the growing season (date of measurement) and agronomic traits for wheat grown in multiple yield trials (1-10) in Jamalpur, Bangladesh

**Traits**	**Trial_1**	**Trial_2**	**Trial_3**	**Trial_4**	**Trial_5**	**Trial_6**	**Trial_7**	**Trial_8**	**Trial_9**	**Trial_10**
CT_20160123	0.06	0.00	-0.21	-0.15	-0.16	0.00	0.00	0.12	0.12	0.12
CT_20160204	-0.03	-0.27 *	0.05	0.02	-0.04	0.07	-0.33 *	0.17	-0.04	0.14
CT_20160212	-0.25	0.05	0.04	-0.19	-0.13	0.15	-0.23	0.26 *	-0.10	-0.29 *
CT_20160223	0.09	-0.16	-0.29 *	-0.03	0.13	0.15	0.11	0.08	-0.06	-0.09
CT_20160228	-0.20	-0.22	-0.39 **	0.11	-0.25	-0.18	-0.02	0.09	0.04	-0.12
CT_20160302	-0.54 ***	-0.50 ***	-0.58 ***	-0.33 *	-0.46 ***	-0.35 **	-0.13	-0.04	0.00	-0.08
CT_20160309	-0.14	-0.21	-0.17	-0.21	-0.31 *	-0.04	-0.20	-0.26 *	-0.39 **	-0.23
CT_20160315	-0.02	0.21	-0.10	0.00	-0.15	0.01	-0.02	-0.09	-0.12	0.05
NDVI_20160121	0.24	0.33 **	0.23	-0.05	0.43 ***	-0.28 *	0.51 ***	0.03	0.29 *	0.16
NDVI_20160130	0.20	0.28 *	0.15	0.02	0.35 **	-0.16	0.31 *	-0.09	0.28 *	0.17
NDVI_20160203	0.32 *	0.29 *	0.24	-0.06	0.13	-0.36 **	0.29 *	0.03	0.26 *	0.58 ***
NDVI_20160207	0.23	-0.18	0.27 *	0.13	0.06	-0.02	0.33 *	-0.13	-0.10	-0.23
NDVI_20160223	0.47 ***	0.06	0.18	-0.24	-0.10	-0.02	0.02	-0.13	0.02	0.00
NDVI_20160228	0.30 *	0.46 ***	0.49 ***	0.38 **	0.28 *	0.34 **	0.58 ***	0.02	0.28 *	0.37 **
NDVI_20160303	0.34 **	0.40 **	0.49 ***	0.45 ***	0.48 ***	0.54 ***	0.45 ***	0.07	0.44 ***	0.17
NDVI_20160310	0.07	0.13	0.09	0.11	0.04	0.28 *	0.34 **	0.00	0.21	0.16
NDVI_20160315	-0.12	0.03	-0.05	-0.03	-0.21	0.14	-0.07	0.19	-0.04	0.07
Days to Heading	0.04	-0.03	0.04	-0.09	-0.24	0.00	0.05	0.06	-0.05	-0.21
Days to Maturity	0.28 *	0.29 *	0.29 *	0.26 *	0.19	0.34 **	0.28 *	0.04	0.37 **	0.00
Plant Height	0.13	0.19	0.27 *	0.33 *	0.30 *	0.46 ***	0.10	0.03	0.32 *	0.45 ***
Spike Number	-0.03	0.19	0.21	-0.03	0.16	0.03	0.4 **	-0.37 **	-0.04	0.48 ***
Spike length	-0.08	-0.22	-0.28 *	-0.21	0.19	-0.03	0.07	-0.01	0.12	0.01
Spikelets per spike	0.02	0.06	-0.03	0.05	0.28 *	0.33 **	0.03	0.00	0.22	0.09
Grains per spike	0.11	0.02	0.22	0.16	0.08	0.04	0.10	0.06	0.22	0.11
Thousand grain wt.	0.44 ***	0.47 ***	0.51 ***	0.42 ***	0.43 ***	0.45 ***	0.33 **	0.03	0.25	0.33 **

* Significant at the 0.05 probability level.
** Significant at the 0.01 probability level.
*** Significant at the <0.001 probability level.

Supplementary Table 7. Correlation between grain yield and phenotypic traits for the 2016-17 season. Data include normalized difference vegetation index (NDVI) and canopy temperature (CT) measured at multiple times across the growing season (date of measurement) and agronomic traits for wheat grown in multiple yield trials (1-11) in Jamalpur, Bangladesh

Traits	Trial_1	Trial_2	Trial_3	Trial_4	Trial_5	Trial_6	Trial_7	Trial_8	Trial_9	Trial_10	Trial_11
CT_20170104	0.05	-0.30 *	0.16	-0.39 **	-0.32 *	-0.18	-0.02	0.05	-0.29 *	-0.36 **	-0.35 **
CT_20170109	-0.05	0.01	-0.02	-0.35 **	-0.51 ***	-0.15	-0.12	0.21	-0.32 *	-0.33 **	-0.26 *
CT_20170114	-0.02	-0.20	-0.17	-0.29 *	-0.52 ***	-0.21	-0.26 *	0.03	-0.56 ***	-0.45 ***	-0.31 *
CT_20170120	0.05	-0.17	-0.04	-0.41 ***	-0.38 **	0.02	-0.09	0.05	-0.15	-0.12	-0.30 *
CT_20170125	-0.13	-0.06	-0.25	-0.34 **	-0.61 ***	-0.03	-0.12	-0.14	-0.33 **	-0.35 **	-0.08
CT_20170131	-0.11	-0.07	-0.07	-0.09	-0.33 **	-0.10	-0.18	0.13	-0.10	-0.22	-0.21
CT_20170205	-0.26 *	-0.13	-0.39 **	-0.34 **	-0.58 ***	-0.31 *	-0.27 *	-0.31 *	-0.53 ***	-0.52 ***	-0.36 **
CT_20170210	-0.32 *	-0.32 *	-0.13	-0.33 *	-0.62 ***	-0.30 *	-0.15	0.06	-0.38 **	-0.53 ***	-0.28 *
CT_20170215	-0.03	-0.31 *	-0.30 *	-0.25	-0.42 ***	-0.16	-0.15	-0.13	-0.38 **	-0.56 ***	-0.50 ***
CT_20170221	-0.28 *	-0.34 **	-0.36 **	-0.26 *	-0.63 ***	-0.12	-0.20	-0.14	-0.19	-0.47 ***	-0.46 ***
CT_20170225	-0.40 **	-0.31 *	0.05	-0.34 **	-0.45 ***	-0.10	-0.42 ***	-0.11	-0.31 *	-0.53 ***	-0.38 **
CT_20170302	-0.44 ***	-0.26 *	0.04	-0.32 *	-0.37 **	-0.19	-0.36 **	-0.03	-0.17	-0.31 *	-0.30 *
CT_20170307	0.10	-0.4 **	0.16	-0.04	-0.17	0.06	-0.02	0.08	0.02	-0.20	0.00
CT_20170313	-0.28 *	-0.29 *	-0.15	-0.05	-0.11	-0.01	0.01	0.14	0.21	-0.28 *	-0.18
NDVI_20170103	-0.03	0.07	-0.20	0.29 *	0.29 *	0.00	-0.23	-0.21	0.39 **	0.33 *	0.28 *
NDVI_20170108	0.00	0.23	-0.21	0.20	0.30 *	-0.02	-0.28 *	-0.11	0.48 ***	0.40 **	0.40 **
NDVI_20170114	0.00	0.32 *	-0.09	0.25	0.30 *	0.06	-0.14	-0.17	0.50 ***	0.44 ***	0.39 **
NDVI_20170120	0.08	0.23	0.01	0.26 *	0.35 **	0.26 *	-0.15	0.06	0.55 ***	0.47 ***	0.31 *
NDVI_20170125	0.09	0.30 *	-0.09	0.28 *	0.43 ***	0.12	0.11	-0.04	0.55 ***	0.46 ***	0.38 **
NDVI_20170131	-0.07	0.25	0.21	0.29 *	0.41 **	0.23	0.20	0.06	0.62 ***	0.54 ***	0.33 *
NDVI_20170205	0.17	0.23	0.22	0.28 *	0.43 ***	0.25	0.26 *	0.00	0.64 ***	0.56 ***	0.45 ***
NDVI_20170210	-0.01	0.35 **	-0.02	0.15	0.40 **	0.21	0.30 *	-0.07	0.36 **	0.36 **	0.29 *
NDVI_20170215	0.04	0.12	-0.21	-0.09	0.3 *	0.22	0.35 **	0.01	0.52 ***	0.56 ***	0.16
NDVI_20170220	0.12	0.36 **	-0.03	0.25	0.46 ***	0.00	0.30 *	0.10	0.22	0.43 ***	0.03
NDVI_20170225	0.42 ***	0.36 **	-0.16	0.33 **	0.21	0.10	0.30 *	0.03	0.22	0.28 *	0.07
NDVI_20170302	0.3 *	0.18	-0.21	-0.05	-0.10	-0.02	0.28 *	0.12	0.06	0.27 *	0.13
NDVI_20170307	0.07	0.10	-0.35 **	-0.09	-0.11	-0.18	0.08	0.20	-0.04	0.23	0.13
NDVI_20170313	-0.12	0.07	-0.35 **	-0.04	-0.22	-0.23	-0.10	-0.21	-0.26 *	0.14	0.14
Days to Heading	-0.31 *	-0.18	-0.57 ***	-0.25	-0.21	-0.61 ***	-0.28 *	-0.38 **	-0.26 *	-0.11	0.04
Days to Maturity	0.13	0.01	-0.41 **	0.08	-0.11	-0.22	0.08	-0.06	-0.14	0.08	0.01
Plant Height	0.18	0.12	-0.12	0.14	0.29 *	0.15	-0.01	0.18	0.44 ***	0.32 *	0.29 *
Spike Number	0.31 *	0.14	0.02	0.22	0.22	0.02	-0.11	-0.19	0.31 *	0.48 ***	0.35 **
Spike length	-0.01	-0.11	-0.27 *	-0.16	-0.13	-0.23	0.04	-0.12	0.11	0.14	0.11
Spikelets per spike	-0.01	-0.3 *	-0.24	-0.26 *	-0.07	-0.15	0.14	-0.07	0.01	0.09	0.31 *
Grains per spike	0.17	-0.04	-0.02	0.05	0.15	0.08	0.23	-0.13	0.33 *	0.21	0.22
Thousand grain wt.	0.56 ***	0.45 ***	0.53 ***	0.31 *	0.53 ***	0.51 ***	0.48 ***	0.48 ***	0.29 *	0.09	-0.03

* Significant at the 0.05 probability level.
** Significant at the 0.01 probability level.
*** Significant at the <0.001 probability level.

Supplementary Table 8. Correlation between grain yield and phenotypic traits for the 2017-18 season. Data include normalized difference vegetation index (NDVI) and canopy temperature (CT) measured at multiple times across the growing season (date of measurement) and agronomic traits for wheat grown in multiple yield trials (1-11) in Jamalpur, Bangladesh

Traits	Trial_1	Trial_2	Trial_3	Trial_4	Trial_5	Trial_6	Trial_7	Trial_8	Trial_9	Trial_10	Trial_11
CT_20180126	-0.37 **	-0.16	-0.41 **	-0.44 ***	-0.18	-0.57 ***	0.06	-0.29 *	0.08	-0.4 **	-0.51 ***
CT_20180131	-0.36 **	-0.03	-0.38 **	-0.51 ***	-0.06	-0.46 ***	-0.12	-0.06	0.01	-0.48 ***	-0.50 ***
CT_20180205	-0.31 *	-0.22	-0.32 *	-0.30 *	-0.14	-0.6 ***	-0.32 *	-0.40 **	-0.12	-0.45 ***	-0.58 ***
CT_20180210	-0.37 **	-0.28 *	-0.53 ***	-0.32 *	-0.07	-0.73 ***	-0.20	-0.29 *	-0.02	-0.23	-0.50 ***
CT_20180214	-0.43 ***	-0.30 *	-0.39 **	-0.41 **	-0.25	-0.61 ***	-0.28 *	-0.48 ***	-0.07	-0.43 ***	0.00
CT_20180219	-0.36 **	-0.39 **	-0.46 ***	-0.17	0.06	-0.59 ***	-0.15	-0.28 *	0.01	-0.39 **	-0.36 **
CT_20180225	-0.26 *	-0.12	-0.23	-0.21	-0.10	-0.64 ***	0.09	-0.24	-0.03	-0.10	-0.09
CT_20180301	-0.32 *	-0.17	-0.25	-0.19	-0.03	-0.45 ***	-0.11	-0.41 **	-0.30 *	-0.22	-0.28 *
CT_20180305	-0.26 *	-0.20	-0.05	-0.09	-0.12	-0.28 *	0.04	-0.37 **	0.06	-0.12	0.02
CT_20180310	-0.30 *	0.04	-0.32 *	-0.10	0.04	-0.42 ***	0.21	-0.01	0.02	-0.18	-0.17
CT_20180315	-0.24	-0.08	-0.23	0.12	-0.03	-0.12	0.16	0.01	0.13	-0.01	-0.19
CT_20180320	-0.09	-0.25	-0.28 *	0.11	0.06	0.24	0.28 *	0.06	-0.25	0.03	-0.17
NDVI_20180126	0.28 *	0.08	0.50 ***	0.43 ***	0.12	0.17	0.04	0.26 *	-0.07	0.28 *	0.56 ***
NDVI_20180131	0.28 *	0.21	0.38 **	0.42 ***	0.02	0.38 **	0.08	-0.06	-0.1	0.22	0.63 ***
NDVI_20180204	0.27 *	0.19	0.35 **	0.37 **	0.30 *	0.21	0.20	0.23	0.00	0.08	0.59 ***
NDVI_20180210	0.32 *	0.37 **	0.35 **	0.44 ***	0.35 **	0.62 ***	0.22	0.35 **	0.22	0.41 **	0.38 **
NDVI_20180214	0.42 ***	0.27 *	0.36 **	0.21	0.14	0.61 ***	0.29 *	0.27 *	-0.16	0.41 **	0.17
NDVI_20180219	0.34 **	0.00	0.35 **	0.02	0.14	0.58 ***	-0.09	0.47 ***	0.15	0.34 **	0.35 **
NDVI_20180226	0.06	0.09	0.03	-0.23	0.16	0.37 **	0.06	-0.15	-0.07	-0.24	0.19
NDVI_20180301	0.16	0.02	0.24	-0.04	-0.08	0.36 **	0.13	0.15	0.27 *	0.04	-0.03
NDVI_20180305	0.03	-0.06	0.07	0.03	0.19	0.10	0.05	0.08	-0.14	-0.24	0.04
NDVI_20180310	-0.28 *	-0.32 *	-0.03	-0.23	-0.24	-0.18	-0.09	-0.12	-0.18	-0.29 *	-0.03
NDVI_20180315	-0.35 **	-0.32 *	-0.13	-0.23	-0.23	-0.38 **	-0.29 *	-0.27 *	-0.22	-0.26 *	-0.35 **
NDVI_20180320	-0.20	-0.23	-0.29 *	0.01	-0.21	-0.49 ***	-0.25	-0.37 **	-0.26 *	-0.32 *	-0.48 ***
Days to Heading	-0.33 **	-0.45 ***	-0.31 *	-0.42 ***	-0.40 **	-0.5 ***	-0.36 **	-0.23	-0.45 ***	-0.40 **	-0.22
Days to Maturity	-0.19	-0.34 **	-0.08	-0.33 *	-0.25	-0.24	-0.14	-0.12	-0.29 *	-0.16	-0.12
Plant Height	0.31 *	0.12	0.53 ***	0.34 **	0.16	0.33 **	0.19	0.29 *	0.16	-0.13	0.19
Spike Number	0.42 ***	0.09	0.42 ***	0.42 ***	-0.01	0.22	0.09	0.06	0.39 **	0.39 **	0.53 ***
Spike length	0.18	-0.14	0.04	0.13	-0.28 *	0.18	0.15	0.37 **	0.23	0.05	-0.18
Spikelets per spike	0.05	-0.20	0.09	-0.18	-0.11	0.03	0.14	0.19	0.08	0.14	-0.06
Grains per spike	-0.10	0.19	-0.01	0.22	0.31 *	-0.06	0.07	0.33 *	0.00	0.09	-0.05
Thousand grain wt.	0.26 *	0.61 ***	0.45 ***	0.17	0.5 ***	0.39 **	0.45 ***	0.29 *	0.05	0.20	0.28 *

* Significant at the 0.05 probability level.
** Significant at the 0.01 probability level.
*** Significant at the <0.001 probability level.

Supplementary Table 9. Correlation between grain yield and phenotypic traits for the 2018-19 season. Data include normalized difference vegetation index (NDVI) and canopy temperature (CT) measured at multiple times across the growing season (date of measurement) and agronomic traits for wheat grown in multiple yield trials (1-10) in Jamalpur, Bangladesh

Traits	Trial_1	Trial_2	Trial_3	Trial_4	Trial_5	Trial_6	Trial_7	Trial_8	Trial_9	Trial_10
CT_20190123	-0.21	-0.24	-0.68 ***	-0.47 ***	-0.22	-0.40 **	-0.49 ***	-0.63 ***	-0.52 ***	-0.54 ***
CT_20190127	-0.26 *	-0.37 **	-0.76 ***	-0.45 ***	-0.37 **	-0.36 **	-0.62 ***	-0.68 ***	-0.53 ***	-0.54 ***
CT_20190131	-0.27 *	0.01	-0.30 *	-0.15	-0.16	-0.27 *	-0.18	0.09	-0.08	-0.08
CT_20190205	-0.15	0.02	-0.46 ***	0.10	-0.23	-0.08	-0.28 *	-0.48 ***	-0.50 ***	-0.30 *
CT_20190211	0.15	-0.14	-0.24	0.14	-0.37 **	-0.51 ***	-0.45 ***	-0.55 ***	-0.52 ***	-0.66 ***
CT_20190218	-0.22	0.03	-0.21	-0.11	0.10	-0.6 ***	-0.43 ***	-0.55 ***	-0.63 ***	-0.65 ***
CT_20190223	-0.07	-0.45 ***	0.04	0.02	-0.11	-0.33 *	0.18	-0.04	-0.11	0.11
CT_20190301	-0.13	-0.29 *	0.03	0.02	0.15	-0.31 *	0.30 *	0.13	-0.21	0.17
CT_20190305	-0.28 *	-0.22	-0.19	-0.29 *	-0.17	-0.31 *	-0.4 **	-0.36 **	-0.33 *	-0.53 ***
CT_20190311	0.00	-0.16	0.18	-0.44 ***	0.09	-0.27 *	-0.25	-0.60 ***	-0.06	-0.27 *
CT_20190316	0.10	-0.26 *	-0.24	-0.5 ***	-0.25	-0.01	-0.25	-0.32 *	-0.27 *	-0.22
CT_20190320	0.23	0.08	-0.02	-0.29 *	-0.08	0.01	0.04	-0.11	-0.24	-0.31 *
CT_20190325	-0.14	-0.20	0.42 ***	0.17	0.32 *	0.14	0.10	0.17	0.05	-0.06
NDVI_20190121	0.35 **	0.13	0.66 ***	0.57 ***	0.25	0.34 **	0.32 *	0.59 ***	0.47 ***	0.51 ***
NDVI_20190127	0.25	0.13	0.57 ***	0.56 ***	0.33 **	0.43 ***	0.43 ***	0.65 ***	0.47 ***	0.47 ***
NDVI_20190131	0.29 *	0.30 *	0.24	0.36 **	0.18	0.25	0.25	0.05	-0.01	0.24
NDVI_20190205	0.28 *	0.24	-0.01	0.45 ***	0.12	0.31 *	0.34 **	0.43 ***	0.38 **	0.18
NDVI_20190211	0.41 **	0.21	0.69 ***	0.46 ***	0.29 *	0.53 ***	0.55 ***	0.75 ***	0.62 ***	0.53 ***
NDVI_20190218	0.21	0.37 **	0.69 ***	0.4 **	0.04	0.56 ***	0.40 **	0.74 ***	0.68 ***	0.52 ***
NDVI_20190222	0.12	0.04	-0.02	-0.24	0.09	-0.3 *	0.13	-0.01	0.06	-0.06
NDVI_20190228	0.07	0.18	0.30 *	0.23	-0.10	-0.02	0.08	-0.10	0.05	0.35 **
NDVI_20190305	0.03	0.24	0.38 **	0.39 **	0.25	0.14	0.06	0.58 ***	0.38 **	0.29 *
NDVI_20190311	-0.15	0.18	0.06	0.28 *	0.10	0.06	-0.01	0.28 *	0.07	0.17
NDVI_20190315	-0.19	-0.11	-0.12	-0.10	-0.12	-0.15	-0.25	0.07	-0.10	-0.04
NDVI_20190320	-0.34 **	-0.30 *	-0.37 **	-0.24	-0.22	-0.29 *	-0.43 ***	-0.07	-0.24	-0.16
NDVI_20190325	-0.32 *	-0.09	-0.52 ***	-0.23	-0.24	0.20	-0.09	-0.23	-0.28 *	-0.15
Days to Heading	-0.33 *	-0.05	-0.37 **	-0.10	-0.08	-0.20	-0.37 **	-0.26 *	-0.35 **	-0.20
Days to Maturity	-0.24	0.10	-0.22	0.04	0.03	-0.12	-0.36 **	-0.18	-0.31 *	-0.12
Plant Height	0.42 ***	0.35 **	0.5 ***	0.47 ***	0.26 *	0.20	0.06	0.23	0.02	0.01
Spike Number	0.16	0.13	0.43 ***	0.08	0.23	0.33 *	0.39 **	0.06	0.48 ***	0.17
Spike length	-0.04	-0.04	0.05	0.25	-0.09	0.09	0.03	0.19	0.46 ***	0.12
Spikelets per spike	-0.01	-0.23	-0.12	0.10	-0.02	-0.26 *	-0.31 *	0.14	0.16	-0.12
Grains per spike	-0.01	0.19	0.33 *	0.14	0.26 *	-0.01	0.23	0.06	0.21	-0.04
Thousand grain wt.	0.27 *	0.21	0.07	0.14	0.11	0.37 **	0.03	0.02	0.09	0.01

* Significant at the 0.05 probability level.
** Significant at the 0.01 probability level.
*** Significant at the <0.001 probability level.

Supplementary Table 10. Correlation between grain yield and phenotypic traits for the 2019-20 season. Data include normalized difference vegetation index (NDVI) and canopy temperature (CT) measured at multiple times across the growing season (date of measurement) and agronomic traits for wheat grown in multiple yield trials (1-11) in Jamalpur, Bangladesh

Traits	Trial_1	Trial_2	Trial_3	Trial_4	Trial_5	Trial_6	Trial_7	Trial_8	Trial_9	Trial_10	Trial_11
CT_20200112	-0.44 ***	0.01	-0.01	-0.11	-0.18	-0.07	-0.15	-0.19	-0.23	-0.23	-0.63 ***
CT_20200116	-0.11	-0.08	-0.24	-0.05	0.09	-0.09	-0.32 *	-0.21	-0.15	-0.03	-0.44 ***
CT_20200121	-0.28 *	0.05	-0.12	0.05	-0.20	-0.05	-0.13	-0.09	-0.17	-0.18	-0.48 ***
CT_20200126	-0.13	-0.15	-0.40 **	-0.25	0.12	0.10	-0.17	0.13	-0.20	-0.16	-0.46 ***
CT_20200130	-0.44 ***	-0.12	-0.07	-0.35 **	0.13	0.04	0.08	0.00	-0.22	0.12	-0.24
CT_20200205	-0.12	-0.29 *	-0.35 **	-0.43 ***	-0.28 *	-0.15	0.06	0.10	-0.06	-0.18	-0.31 *
CT_20200210	-0.44 ***	-0.53 ***	-0.42 ***	-0.49 ***	-0.22	-0.06	0.08	0.00	-0.22	-0.26 *	-0.34 **
CT_20200215	-0.08	0.00	-0.13	-0.25	-0.12	-0.04	0.22	-0.23	-0.31 *	-0.12	-0.13
CT_20200220	-0.07	-0.27 *	-0.24	-0.29 *	0.05	0.06	0.02	-0.17	-0.13	-0.27 *	-0.23
CT_20200226	-0.24	-0.12	-0.27 *	-0.20	-0.04	0.08	0.06	0.06	-0.21	-0.22	-0.26 *
CT_20200302	-0.42 ***	-0.29 *	-0.29 *	-0.32 *	-0.19	0.11	-0.01	-0.17	-0.24	-0.28 *	-0.36 **
CT_20200308	-0.18	-0.13	-0.09	-0.36 **	-0.17	0.15	0.07	-0.05	-0.40 **	-0.28 *	-0.36 **
CT_20200313	-0.21	0.07	-0.29 *	-0.17	-0.04	-0.11	-0.03	-0.08	-0.47 ***	-0.28 *	-0.42 ***
CT_20200318	0.08	-0.32 *	-0.22	-0.14	-0.02	-0.28 *	0.25	-0.03	-0.33 *	-0.17	-0.09
CT_20200323	0.23	-0.21	-0.11	-0.16	0.05	0.00	0.18	0.08	-0.20	-0.14	-0.18
NDVI_20200112	0.41 **	0.25	0.35 **	0.27 *	0.15	0.07	0.32 *	0.32 *	0.28 *	-0.01	0.56 ***
NDVI_20200116	0.16	0.22	0.18	0.08	0.14	0.07	0.04	0.29 *	0.10	0.14	0.65 ***
NDVI_20200121	0.37 **	0.30 *	0.50 ***	0.29 *	0.05	-0.07	0.21	0.55 ***	0.26 *	0.08	0.64 ***
NDVI_20200126	0.38 **	0.06	0.21	0.38 **	0.18	-0.11	0.05	0.30 *	0.14	0.18	0.38 **
NDVI_20200130	0.40 **	0.45 ***	0.33 *	0.35 **	0.21	0.03	0.25	0.31 *	0.26 *	0.22	0.65 ***
NDVI_20200205	0.45 ***	0.5 ***	0.29 *	0.31 *	0.45 ***	0.07	0.11	0.27 *	0.16	0.40 **	0.45 ***
NDVI_20200210	0.38 **	0.32 *	0.40 **	0.41 **	0.06	0.05	0.20	0.29 *	0.13	0.36 **	0.17
NDVI_20200215	0.24	0.35 **	0.28 *	0.68 ***	0.36 **	-0.11	0.06	0.16	0.32 *	0.37 **	0.29 *
NDVI_20200220	0.41 **	0.25	0.18	0.53 ***	0.21	0.01	0.42 ***	0.14	0.06	0.18	0.45 ***
NDVI_20200226	0.24	0.33 **	0.36 **	0.41 **	0.28 *	-0.22	0.35 **	0.35 **	0.25	0.37 **	0.57 ***
NDVI_20200302	0.14	0.42 ***	0.31 *	0.37 **	0.32 *	-0.29 *	0.38 **	0.37 **	0.32 *	0.34 **	0.31 *
NDVI_20200308	0.02	0.45 ***	0.13	0.19	0.13	-0.07	0.03	0.23	0.26 *	0.45 ***	0.23
NDVI_20200313	0.00	0.05	0.08	-0.13	0.00	-0.11	-0.34 **	0.10	0.14	0.19	0.07
NDVI_20200318	-0.19	-0.06	-0.05	-0.09	-0.04	-0.21	-0.49 ***	-0.03	-0.11	0.16	0.21
NDVI_20200323	-0.33 *	-0.09	-0.33 **	-0.20	0.03	0.11	-0.60 ***	-0.10	-0.02	0.07	-0.43 ***
GrndCov_20200112	0.28 *	0.16	0.45 ***	0.39 **	0.43 ***	0.02	0.12	0.44 ***	0.41 **	0.25	0.72 ***
GrndCov_20200206	0.29 *	0.24	0.25	0.45 ***	0.46 ***	0.14	0.07	0.39 **	0.42 ***	0.17	0.73 ***
DLA_Feb26	-0.22	-0.11	-0.20	-0.22	0.07	-0.24	0.38 **	0.09	-0.05	-0.31 *	-0.05
DLA_Mar09	0.14	-0.26 *	-0.02	-0.28 *	-0.02	-0.22	0.27 *	0.09	-0.06	-0.03	0.19
Days to Heading	-0.19	-0.03	-0.20	-0.41 **	-0.27 *	-0.46 ***	-0.62 ***	-0.03	-0.25	-0.08	0.12
Days to Maturity	0.07	0.11	-0.01	-0.32 *	-0.15	-0.03	-0.43 ***	0.08	0.06	0.08	0.20
Plant Height	0.42 ***	0.29 *	0.47 ***	0.34 **	0.20	0.19	0.44 ***	0.18	0.32 *	0.07	0.33 **
Spike Number	0.19	0.29 *	0.11	0.44 ***	0.25 *	0.15	0.37 **	0.44 ***	0.44 ***	0.25	0.63 ***
Spikelets per spike	0.21	0.20	0.21	-0.10	0.04	0.03	-0.09	-0.06	-0.07	-0.12	0.23
Grains per spike	0.06	0.35 **	0.31 *	0.21	0.14	0.01	0.11	0.12	-0.10	0.34 **	0.05
Thousand grain wt.	0.19	0.24	0.44 ***	0.42 ***	0.28 *	0.51 ***	0.33 *	0.16	0.28 *	0.07	-0.09

* Significant at the 0.05 probability level.
** Significant at the 0.01 probability level.
*** Significant at the <0.001 probability level.

Supplementary Table 11: Combination of variables in stepwise regression model in five wheat growing seasons from 2016 to 2020.

Seasons	Combination of variables in Stepwise regression model	Comments
2016	GRYLD ~ CT_20160204 + CT_20160223 + CT_20160302 + NDVI_20160121 + NDVI_20160130 + NDVI_20160203 + NDVI_20160207 + NDVI_20160223 + NDVI_20160228 + NDVI_20160303 + DTHD + DAYSMT + PH + SN + SPKLNG + GRNSPK + TGW	3CTs out of 8 and 7NDVIs out of 9
2017	GRYLD ~ CT_20170104 + CT_20170114 + CT_20170131 + CT_20170205 + CT_20170210 + CT_20170221 + CT_20170225 + CT_20170302 + CT_20170307 + CT_20170313 + NDVI_20170108 + NDVI_20170131 + NDVI_20170225 + NDVI_20170302 + DTHD + DAYSMT + PH + SPKLNG + GRNSPK + TGW	10CTs out of 14 and 4NDVIs out of 14
2018	GRYLD ~ CT_20180126 + CT_20180301 + CT_20180310 + NDVI_20180126 + NDVI_20180204 + NDVI_20180214 + NDVI_20180301 + NDVI_20180315 + NDVI_20180320 + DTHD + DAYSMT + PH + SN + GRNSPK + TGW	3CTs out of 12 and 6NDVIs out of 12
2019	GRYLD ~ CT_20190123 + CT_20190127 + CT_20190131 + CT_20190316 + NDVI_20190121 + NDVI_20190205 + NDVI_20190211 + NDVI_20190218 + NDVI_20190305 + NDVI_20190311 + NDVI_20190320 + NDVI_20190325 + DTHD + PH + SN + SPLN + GRNSPK + TGW	4CTs out of 13 and 8NDVIs out of 13
2020	GRYLD ~ CT_20200210 + CT_20200308 + CT_20200313 + CT_20200318 + CT_20200323 + NDVI_20200121 + NDVI_20200205 + NDVI_20200226 + NDVI_20200302 + NDVI_20200308 + NDVI_20200318 + DTHD + DAYSMT + PH + SN + GRNSPK + TGW	5CTs out of 15 and 6NDVIs out of 15

Supplementary Table 12: Selected lines from each year.

YEAR	TRIAL	PLOT	GID	DTHD	DAYSMT	PH	SN	SPLN	GRNSPK	TGW	GRYLD
**2016**	5	entry5051	7046872	68.5	104.9	109.3	307.9	16.1	43.1	45.3	4.40
**2016**	9	entry9040	7175897	73.6	105.6	96.1	325.0	18.4	49.1	34.7	4.03
**2016**	5	entry5001	7171325	62.2	103.0	99.5	204.2	18.5	55.9	37.8	3.91
**2016**	6	entry6028	7174167	67.1	105.6	103.7	353.0	19.1	38.9	43.8	3.86
**2016**	5	entry5041	7173767	72.0	106.9	107.4	353.2	15.2	42.2	34.3	3.78
**2016**	9	entry9030	7175853	69.4	103.6	101.6	318.0	19.8	47.6	33.9	3.73
**2016**	5	entry5050	7173922	70.3	104.0	92.9	312.8	16.1	40.9	36.3	3.70
**2016**	4	entry4012	7177875	71.7	107.9	98.2	288.3	17.0	56.0	31.7	3.65
**2016**	5	entry5037	7173722	70.9	105.4	105.2	291.0	17.2	40.1	41.9	3.64
**2016**	5	entry5003	7171329	71.2	106.5	106.0	276.2	15.3	48.1	37.7	3.63
**2016**	5	entry5054	6333158	65.3	106.0	99.7	352.0	16.0	45.7	33.4	3.62
**2016**	9	entry9025	7175837	71.1	103.5	102.2	263.0	21.3	61.7	31.4	3.61
**2016**	5	entry5030	7173536	71.5	106.4	107.0	320.5	16.7	46.7	31.9	3.58
**2016**	3	entry3052	7177666	73.1	107.5	102.8	215.9	17.9	48.2	41.7	3.56
**2016**	5	entry5031	7046418	71.5	107.3	93.2	335.3	14.3	38.9	33.4	3.54
**2016**	5	entry5048	7173851	69.9	104.5	106.1	340.5	16.3	45.8	34.6	3.51
**2016**	6	entry6024	7047297	65.5	103.7	105.1	277.8	16.0	47.6	38.3	3.51
**2016**	6	entry6031	7174267	74.1	106.0	93.1	282.8	18.4	43.1	41.9	3.51
**2016**	5	entry5059	7047125	67.5	102.5	108.3	289.0	16.8	47.7	33.5	3.50
**2016**	5	entry5002	0	70.4	106.1	106.9	274.5	15.7	46.7	47.9	3.49
**2016**	6	entry6006	7174105	68.9	105.8	101.1	281.8	18.1	41.2	46.0	3.48
**2016**	6	entry6016	7174130	68.4	106.0	98.5	238.8	19.3	49.7	37.8	3.48
**2016**	4	entry4046	7171133	75.7	107.4	96.0	299.9	16.7	51.8	38.7	3.47
**2016**	6	entry6032	7174269	70.5	105.7	100.7	259.7	18.1	48.1	39.0	3.47
**2016**	9	entry9048	7048441	69.3	104.0	85.9	271.0	15.3	51.0	36.0	3.46
**2016**	5	entry5027	7046390	68.7	106.4	100.2	325.9	16.4	44.4	31.9	3.43
**2016**	5	entry5038	7173723	71.4	107.6	92.1	240.1	16.2	44.2	46.0	3.43
**2016**	5	entry5043	7173803	67.8	106.0	98.6	290.3	19.6	50.4	38.1	3.43
**2016**	5	entry5055	7047009	68.2	101.9	106.2	309.4	14.1	44.5	35.9	3.43
**2016**	5	entry5019	7173500	66.8	100.6	104.1	312.5	17.0	39.3	38.5	3.41
**2016**	6	entry6046	7174321	73.1	105.6	107.8	296.3	17.9	51.1	29.8	3.40
**2016**	5	entry5028	7173528	69.6	105.9	100.9	351.1	17.5	42.3	33.8	3.38

**2016**	9	entry9001	7047937	69.4	102.6	103.8	249.0	17.3	49.3	36.9	3.38
**2016**	5	entry5020	7173511	66.8	100.6	98.1	324.0	16.4	42.8	43.0	3.37
**2016**	5	entry5023	7173521	73.9	107.6	92.0	279.3	16.2	47.2	35.2	3.37
**2016**	6	entry6035	7174271	74.4	106.0	105.1	381.5	18.3	43.1	39.0	3.35
**2016**	9	entry9002	0	69.0	105.2	103.3	261.5	18.6	53.9	44.2	3.35
**2016**	5	entry5015	7173488	70.5	106.4	106.3	265.5	16.3	43.9	29.4	3.34
**2016**	5	entry5033	5398530	76.7	107.8	109.8	337.4	15.8	37.4	35.0	3.34
**2016**	4	entry4051	7046099	75.3	108.0	98.4	312.6	20.1	52.7	31.0	3.33
**2016**	9	entry9029	7175852	69.4	102.6	99.8	268.5	16.4	45.5	28.7	3.33
**2016**	4	entry4020	7170548	74.1	107.9	105.5	293.5	17.1	44.0	36.3	3.32
**2016**	6	entry6015	7047225	67.5	105.8	105.2	297.8	16.5	46.8	31.5	3.32
**2016**	6	entry6002	0	70.8	106.0	101.5	364.3	16.7	46.3	45.7	3.31
**2016**	5	entry5005	7173426	70.9	103.6	104.9	345.5	12.9	34.0	30.4	3.28
**2016**	5	entry5053	7174031	71.3	106.4	104.7	272.2	15.5	46.8	36.7	3.28
**2016**	9	entry9020	7175794	69.5	103.4	96.4	315.5	16.9	55.8	33.7	3.27
**2016**	3	entry3051	7177664	71.2	104.0	98.4	240.1	13.4	34.8	40.8	3.24
**2016**	5	entry5016	7173494	69.4	103.2	103.2	289.0	16.5	46.1	33.0	3.24
**2016**	5	entry5022	7173519	70.1	105.2	96.4	266.6	14.6	47.1	46.9	3.22
**2016**	5	entry5029	7173534	71.4	106.0	107.3	275.4	16.8	48.9	28.5	3.22
**2016**	4	entry4009	7177872	72.9	107.1	96.7	271.7	17.8	47.4	37.9	3.21
**2016**	6	entry6029	7174195	74.5	105.0	100.8	256.7	17.1	50.2	21.8	3.21
**2016**	6	entry6053	7174342	70.0	106.1	95.5	338.6	16.7	48.4	32.7	3.21
**2016**	6	entry6056	7174347	69.6	105.8	105.0	245.8	17.1	48.4	34.0	3.20
**2016**	5	entry5014	7173484	72.0	106.9	99.8	355.0	16.6	48.8	30.7	3.19
**2016**	3	entry3055	7177673	73.4	107.4	97.8	231.3	15.4	43.0	39.9	3.18
**2016**	4	entry4041	7171098	70.3	106.3	94.3	312.7	17.3	50.3	35.8	3.18
**2016**	6	entry6050	7174332	71.5	106.2	103.3	221.2	19.0	53.0	32.2	3.18
**2016**	4	entry4060	7046215	66.2	104.2	92.3	232.4	16.7	53.0	37.7	3.17
**2017**	1	entry1051	7399180	67.4	111.5	101.0	440.1	15.7	43.6	35.8	4.69
**2017**	2	entry2049	7399623	69.4	110.3	103.9	346.2	17.0	51.2	34.2	4.64
**2017**	3	entry3059	7400239	68.2	111.9	98.5	328.3	17.7	56.1	35.6	4.60
**2017**	2	entry2046	7399616	69.8	110.4	97.8	414.9	16.1	44.6	35.8	4.56
**2017**	3	entry3036	7399947	61.4	109.1	99.0	407.6	15.9	53.6	37.8	4.56
**2017**	9	entry9048	7398471	66.5	107.0	101.8	370.0	15.1	41.4	37.7	4.55
**2017**	2	entry2041	7399609	61.7	104.2	102.9	325.9	13.7	47.5	43.0	4.48
**2017**	7	entry7002	0	62.5	108.5	105.6	372.0	17.3	41.2	46.6	4.46

**2017**	7	entry7026	6175067	67.2	110.0	108.6	252.2	19.2	58.1	39.5	4.46
**2017**	4	entry4045	7400456	65.3	108.7	102.0	437.4	18.5	65.9	32.5	4.45
**2017**	3	entry3035	7399946	67.5	104.8	97.5	336.7	16.9	52.8	40.4	4.43
**2017**	7	entry7016	7311105	68.1	109.5	101.9	367.1	17.6	54.4	40.2	4.40
**2017**	3	entry3026	6175067	67.9	109.4	104.0	293.9	20.8	52.5	40.1	4.39
**2017**	9	entry9029	7398428	67.5	107.5	109.5	247.1	18.5	52.2	45.2	4.37
**2017**	8	entry8032	7397762	68.0	107.8	104.7	297.3	16.8	57.7	46.7	4.30
**2017**	11	entry11037	7174105	69.9	109.0	98.7	250.7	19.1	43.1	50.0	4.30
**2017**	7	entry7042	7397189	75.9	112.5	120.6	280.2	19.9	46.6	36.3	4.29
**2017**	5	entry5040	7396142	70.0	112.3	107.2	365.5	18.5	56.8	41.8	4.29
**2017**	7	entry7008	7310918	70.8	112.0	102.2	245.7	17.6	55.8	44.7	4.28
**2017**	6	entry6041	7396710	68.9	109.5	105.6	328.4	18.2	46.3	39.0	4.27
**2017**	8	entry8002	0	65.4	109.6	109.5	324.0	17.8	47.4	44.3	4.26
**2017**	3	entry3042	7400069	63.0	106.2	106.0	319.2	17.2	44.3	42.2	4.23
**2017**	3	entry3050	7400194	62.8	103.8	99.0	412.1	17.3	40.9	40.4	4.23
**2017**	8	entry8004	7397520	63.0	109.2	105.0	383.3	20.4	61.4	40.5	4.22
**2017**	4	entry4020	7400308	61.0	107.2	96.0	364.3	17.0	50.1	34.7	4.21
**2017**	5	entry5050	7396176	70.0	111.7	101.9	309.4	16.6	46.7	42.4	4.21
**2017**	7	entry7020	7396931	69.0	110.0	103.8	391.6	19.9	55.7	37.1	4.21
**2017**	11	entry11051	7175837	72.1	109.3	112.6	267.7	19.3	47.9	42.0	4.20
**2017**	5	entry5036	7396135	68.5	109.9	96.7	343.9	17.0	39.3	40.6	4.20
**2017**	8	entry8053	7398192	68.0	110.7	108.9	304.2	19.3	49.1	35.9	4.20
**2017**	4	entry4044	7400453	66.4	107.6	103.0	273.6	18.0	57.1	32.5	4.19
**2017**	1	entry1050	7399179	66.0	108.1	101.0	432.1	17.6	48.3	36.6	4.19
**2017**	7	entry7033	6332122	69.3	111.0	105.0	324.0	19.4	52.0	41.3	4.18
**2017**	4	entry4056	7400488	57.3	105.3	89.5	379.2	15.6	43.4	38.7	4.16
**2017**	4	entry4022	7400313	59.0	106.1	99.5	388.7	16.8	46.6	40.9	4.15
**2017**	3	entry3038	7399956	60.7	107.0	103.5	427.6	18.8	55.6	35.0	4.15
**2017**	2	entry2006	7399442	69.5	109.8	101.6	395.9	16.7	54.4	35.6	4.14
**2017**	8	entry8056	7398211	70.3	110.3	110.0	316.4	20.3	56.3	43.9	4.14
**2017**	7	entry7005	7310902	71.0	109.0	99.6	299.0	21.6	60.4	35.8	4.12
**2017**	2	entry2048	7399621	67.7	109.9	99.6	437.1	15.9	43.7	35.1	4.12
**2017**	3	entry3037	7399950	68.5	107.8	94.5	370.1	18.5	56.0	35.8	4.12
**2017**	8	entry8038	7397893	67.5	106.0	106.3	306.7	18.8	52.9	39.1	4.11
**2017**	3	entry3016	7399823	70.6	111.4	99.0	298.6	18.6	59.5	39.0	4.10
**2017**	3	entry3057	7313697	72.8	113.6	101.5	327.2	18.2	40.0	40.4	4.10

**2017**	9	entry9012	7398326	69.5	110.5	108.5	357.3	18.8	53.4	39.2	4.10
**2017**	7	entry7036	7311300	74.3	111.0	112.4	314.7	21.0	57.5	36.2	4.09
**2017**	4	entry4019	7400306	62.5	107.7	102.5	285.3	16.3	47.5	36.7	4.08
**2017**	10	entry10005	7400872	65.7	108.5	93.6	339.7	18.5	46.1	46.0	4.07
**2017**	9	entry9003	7398243	63.5	104.0	107.1	315.2	17.0	48.3	40.3	4.05
**2017**	8	entry8043	7398014	67.5	108.9	101.4	334.3	19.3	48.2	38.5	4.04
**2017**	8	entry8001	7397501	62.5	107.6	106.6	308.4	18.0	46.4	37.9	4.04
**2017**	7	entry7041	7397183	70.6	116.0	105.1	315.8	18.5	51.1	40.0	4.02
**2017**	7	entry7021	7311134	68.7	110.0	106.3	282.3	21.4	69.2	38.4	4.02
**2017**	7	entry7015	7311059	64.4	108.5	103.9	309.4	15.7	41.0	43.0	4.01
**2017**	4	entry4026	6175067	65.7	111.2	101.0	338.0	18.3	56.5	38.4	4.00
**2017**	9	entry9031	7398433	67.0	108.5	110.8	345.5	19.7	49.8	40.4	4.00
**2017**	4	entry4050	7400466	70.4	111.1	98.5	365.5	19.5	57.7	33.8	3.99
**2017**	2	entry2045	7399615	67.9	108.8	97.4	389.2	17.2	51.0	32.2	3.99
**2017**	10	entry10039	6341870	75.3	114.0	95.8	312.5	16.0	47.3	39.8	3.99
**2017**	9	entry9005	7398254	71.0	109.0	106.4	256.9	18.1	51.2	43.8	3.98
**2018**	10	entry10001	7631563	67.0	107.5	82.0	371.6	16.8	46.3	42.9	4.78
**2018**	4	entry4042	7626450	68.4	111.2	98.5	328.1	15.8	50.6	40.8	4.24
**2018**	4	entry4044	7626460	64.5	104.8	89.9	290.2	15.7	49.8	41.3	4.06
**2018**	9	entry9016	7631006	77.4	111.5	94.5	347.6	19.4	57.8	44.0	4.06
**2018**	8	entry8031	7629982	77.2	113.5	95.3	254.1	18.4	57.4	45.3	3.96
**2018**	9	entry9033	6332122	73.3	111.0	89.5	266.7	16.7	46.4	45.5	3.91
**2018**	8	entry8039	6341870	77.1	112.9	88.4	243.1	19.3	59.2	43.5	3.89
**2018**	10	entry10041	7632416	72.1	108.4	86.0	229.5	18.2	48.5	45.3	3.86
**2018**	10	entry10016	7631846	68.5	108.8	90.3	240.0	18.7	41.0	51.2	3.85
**2018**	8	entry8060	7630530	73.5	111.0	87.4	256.9	17.0	41.4	48.5	3.85
**2018**	8	entry8046	7630341	77.3	112.5	100.2	283.2	23.2	63.6	43.5	3.80
**2018**	9	entry9046	7631434	73.2	110.5	91.0	378.5	20.4	57.8	45.0	3.80
**2018**	3	entry3045	7626319	69.6	109.2	98.3	299.0	13.9	49.4	50.8	3.80
**2018**	10	entry10009	7631640	76.5	110.1	86.7	296.6	17.6	52.9	44.9	3.79
**2018**	8	entry8016	7629772	75.7	111.5	91.0	299.0	18.3	57.6	47.5	3.79
**2018**	6	entry6005	7627721	74.1	111.0	95.2	230.5	20.6	56.3	42.8	3.77
**2018**	9	entry9006	7630704	78.1	111.5	86.5	303.7	19.8	45.2	35.3	3.74
**2018**	6	entry6015	7627903	67.3	110.5	90.3	212.5	17.3	57.6	43.5	3.74
**2018**	8	entry8040	7630179	70.5	112.0	92.5	264.9	17.8	52.7	50.3	3.72
**2018**	10	entry10048	7632462	70.5	110.9	91.4	232.3	17.6	47.6	45.2	3.72

**2018**	10	entry10005	7631608	79.0	113.6	87.5	248.9	17.1	41.1	40.9	3.71
**2018**	4	entry4050	7626569	80.5	114.6	97.6	239.4	19.3	51.0	37.7	3.70
**2018**	4	entry4052	7626572	70.9	111.8	97.3	217.4	19.9	55.0	46.8	3.68
**2018**	10	entry10026	6175067	72.4	111.2	88.3	244.6	19.5	51.4	48.6	3.67
**2018**	8	entry8032	7630037	77.3	112.5	98.6	297.2	16.4	58.9	43.3	3.67
**2018**	3	entry3005	7626072	77.1	112.6	94.2	243.5	22.6	59.1	40.8	3.66
**2018**	9	entry9030	7631193	69.9	109.0	92.7	278.7	18.9	45.4	44.5	3.66
**2018**	9	entry9018	7631063	72.7	111.5	90.1	220.9	18.0	49.0	41.5	3.65
**2018**	4	entry4031	7626416	75.4	110.6	91.6	194.2	19.2	49.8	47.5	3.65
**2018**	9	entry9009	7630834	69.5	110.0	89.3	320.2	18.1	47.7	44.5	3.64
**2018**	8	entry8007	7629741	69.0	110.5	87.8	287.0	16.9	45.4	45.0	3.63
**2018**	8	entry8030	7629913	76.3	112.4	89.4	278.0	17.1	47.2	40.3	3.62
**2018**	10	entry10006	7631614	80.0	115.9	88.5	228.3	15.6	39.9	41.7	3.62
**2018**	8	entry8011	7629752	70.6	109.4	86.0	239.8	15.8	51.8	45.3	3.62
**2018**	8	entry8015	7629766	72.2	110.5	91.4	306.3	19.9	46.9	47.3	3.60
**2018**	8	entry8035	7630047	76.3	111.0	92.3	236.8	18.9	55.6	51.0	3.60
**2018**	8	entry8005	7629732	71.8	109.0	84.7	270.2	18.2	45.8	45.5	3.59
**2018**	9	entry9015	7630977	71.7	111.5	87.7	265.8	17.7	50.9	41.8	3.59
**2018**	4	entry4043	7626451	75.4	109.7	91.2	301.4	14.5	39.8	38.6	3.59
**2018**	10	entry10043	7632444	70.0	108.1	88.9	272.0	20.3	57.4	39.7	3.58
**2018**	11	entry11029	7399601	78.1	111.0	90.8	314.5	15.4	41.1	45.4	3.57
**2018**	7	entry7031	7629318	75.2	111.5	95.5	284.0	17.8	52.9	41.8	3.56
**2018**	3	entry3041	7626305	77.5	113.1	96.4	223.0	19.4	55.3	50.5	3.56
**2018**	6	entry6032	7628201	73.3	109.5	90.7	188.6	19.1	53.7	46.5	3.55
**2018**	8	entry8057	7630477	76.2	111.0	87.6	265.2	17.6	46.2	47.8	3.54
**2018**	3	entry3015	7626094	71.5	108.9	93.7	269.0	20.6	46.4	43.5	3.52
**2018**	10	entry10004	7631568	72.5	109.0	87.9	360.0	19.8	45.1	39.5	3.51
**2018**	9	entry9008	7630830	69.4	108.5	85.6	302.6	14.5	46.8	45.8	3.51
**2018**	10	entry10045	7632450	69.0	108.7	88.5	237.2	17.0	43.1	51.9	3.50
**2018**	10	entry10003	7631566	75.5	111.5	84.5	242.6	16.6	42.9	43.7	3.50
**2018**	10	entry10023	7631994	68.5	106.4	84.2	264.9	19.2	51.5	48.2	3.50
**2018**	8	entry8048	7630352	77.1	112.0	96.7	299.3	22.4	55.0	42.5	3.50
**2018**	8	entry8026	6175067	71.9	110.0	89.8	245.5	19.6	55.0	46.3	3.50
**2018**	4	entry4060	7626689	77.8	112.7	92.6	316.7	17.2	51.2	45.5	3.49
**2018**	10	entry10029	7632194	71.9	106.8	83.6	267.7	18.7	42.1	39.9	3.47
**2018**	9	entry9048	7631438	73.3	110.0	94.2	271.5	18.0	53.8	43.5	3.47

**2018**	8	entry8038	7630077	75.6	111.9	90.4	343.1	18.8	56.6	39.3	3.46
**2018**	7	entry7009	7628883	70.6	106.0	88.3	208.5	18.7	44.0	51.4	3.46
**2018**	10	entry10010	7631716	77.6	111.3	93.8	269.7	18.3	54.0	39.3	3.45
**2018**	8	entry8047	7630348	75.7	112.0	93.9	266.6	21.2	61.3	45.0	3.44
**2019**	9	entry9055	8053864	79.5	116.3	93.9	169.0	20.6	52.1	47.8	5.25
**2019**	8	entry8035	8052004	75.4	116.0	99.1	156.0	18.5	49.2	40.1	5.16
**2019**	3	entry3020	8051113	73.8	113.9	105.0	251.9	16.4	52.5	40.9	4.97
**2019**	3	entry3058	8044990	74.8	115.2	98.5	182.5	20.0	53.3	42.3	4.94
**2019**	10	entry10034	8054309	80.3	118.0	93.0	149.0	16.2	36.5	44.0	4.88
**2019**	8	entry8025	8051868	72.8	106.0	97.4	148.5	17.5	49.9	48.0	4.62
**2019**	5	entry5028	8047924	74.9	113.0	100.3	169.0	17.5	60.7	48.4	4.59
**2019**	3	entry3034	8051237	81.8	118.2	95.5	193.0	18.1	55.0	43.4	4.57
**2019**	4	entry4039	6681676	72.6	112.7	105.8	163.6	18.3	49.3	46.8	4.41
**2019**	8	entry8007	8051474	77.2	117.1	105.7	176.5	21.5	47.4	41.3	4.40
**2019**	8	entry8009	8051496	80.4	118.6	100.3	166.5	17.6	43.7	42.5	4.39
**2019**	10	entry10026	6341870	79.4	118.0	90.3	144.1	20.2	56.8	44.8	4.37
**2019**	3	entry3039	6681676	71.0	113.0	96.0	172.4	15.3	53.3	44.1	4.36
**2019**	10	entry10046	8054553	70.8	107.0	95.6	158.1	17.0	41.5	49.6	4.36
**2019**	3	entry3047	8044862	78.9	116.7	101.0	185.9	19.7	58.5	42.9	4.36
**2019**	3	entry3050	8044907	74.4	112.6	87.5	203.6	16.1	50.6	41.2	4.35
**2019**	9	entry9045	8053288	78.0	114.6	98.2	194.0	17.1	41.2	46.4	4.32
**2019**	10	entry10060	8055317	79.9	117.0	97.3	163.5	19.5	56.0	44.6	4.30
**2019**	4	entry4017	6175067	74.2	111.6	107.3	149.8	19.9	60.0	48.6	4.28
**2019**	10	entry10039	6681676	72.4	114.0	97.8	136.2	16.0	43.7	42.5	4.27
**2019**	10	entry10005	8054147	79.8	118.0	102.9	141.2	16.0	48.1	43.3	4.26
**2019**	8	entry8003	8051443	74.6	114.9	101.3	160.5	19.3	48.8	49.0	4.26
**2019**	9	entry9002	7890127	66.5	108.4	89.2	162.0	17.3	47.7	52.8	4.26
**2019**	8	entry8002	7890127	66.0	111.0	93.8	154.0	19.0	45.5	49.0	4.25
**2019**	3	entry3048	8044864	80.2	117.8	89.5	207.7	18.7	55.0	38.1	4.24
**2019**	1	entry1015	8048881	80.0	115.7	86.0	175.2	21.1	51.2	37.3	4.23
**2019**	10	entry10035	8054321	79.5	115.0	95.7	169.3	18.3	49.0	49.8	4.18
**2019**	7	entry7039	6681676	73.7	115.1	100.5	154.6	16.7	46.0	41.7	4.16
**2019**	3	entry3011	8051039	78.3	115.5	96.0	105.2	18.6	64.8	41.2	4.14
**2019**	4	entry4056	8046775	76.6	114.4	101.2	168.3	15.7	49.0	53.4	4.13
**2019**	7	entry7049	8059712	72.0	108.1	90.0	161.1	16.9	37.8	44.0	4.13
**2019**	10	entry10036	8054354	73.6	109.5	91.4	171.6	19.4	54.3	46.6	4.12

**2019**	4	entry4055	8046733	75.8	111.4	98.5	136.1	16.0	45.4	50.6	4.11
**2019**	9	entry9057	8053942	76.5	114.1	94.1	155.0	19.0	42.8	51.0	4.08
**2019**	10	entry10014	8054192	81.6	118.0	87.8	168.8	19.3	47.4	45.1	4.08
**2019**	3	entry3017	6175067	75.1	112.5	101.5	161.9	20.0	61.0	47.2	4.07
**2019**	7	entry7011	8058242	81.1	118.0	102.5	166.2	18.7	61.2	45.8	4.07
**2019**	10	entry10043	8054496	75.7	113.5	91.0	161.0	17.4	47.3	44.1	4.06
**2019**	7	entry7022	8059025	75.3	114.5	100.0	171.3	14.7	56.2	39.5	4.05
**2019**	7	entry7038	8059211	77.2	112.1	103.5	154.6	18.8	52.8	47.6	4.04
**2019**	8	entry8026	6341870	80.0	119.0	96.6	136.0	17.2	55.2	45.0	4.03
**2019**	3	entry3040	8044697	76.6	115.0	94.0	198.5	20.9	66.0	48.1	4.03
**2019**	3	entry3010	8051036	77.7	113.7	100.0	175.7	17.8	52.8	42.4	4.03
**2019**	8	entry8010	8051507	81.2	118.4	105.8	136.5	19.5	47.4	38.8	4.03
**2019**	7	entry7005	8058152	74.0	112.0	88.0	161.4	18.2	52.8	49.9	4.00
**2019**	8	entry8023	8051812	75.8	114.5	98.9	153.5	19.9	46.3	42.5	4.00
**2019**	3	entry3051	8044909	76.8	113.2	98.5	182.4	19.7	50.5	45.9	3.99
**2019**	6	entry6032	8057419	80.7	118.1	97.3	190.6	15.0	58.8	45.1	3.99
**2019**	4	entry4046	8046693	82.6	119.0	102.9	152.1	17.7	34.9	40.8	3.99
**2019**	2	entry2026	6341870	77.0	118.2	92.6	258.6	18.6	61.3	49.1	3.94
**2019**	10	entry10031	8054238	81.3	118.5	101.0	158.5	19.5	64.6	47.3	3.94
**2019**	10	entry10025	8054214	81.5	118.5	90.4	152.6	17.0	56.8	42.5	3.93
**2019**	5	entry5016	8047605	73.9	117.0	95.3	195.8	18.3	51.8	42.6	3.92
**2019**	5	entry5032	8048083	78.7	115.7	101.5	156.7	19.9	52.8	48.3	3.91
**2019**	8	entry8030	8051926	74.4	113.1	102.8	162.5	18.7	45.8	50.0	3.90
**2019**	8	entry8001	8051423	81.1	118.9	96.3	146.5	21.2	43.8	42.3	3.88
**2019**	8	entry8016	8051574	78.2	116.9	94.2	163.5	18.4	52.2	45.3	3.87
**2019**	3	entry3054	7175970	74.6	110.7	103.5	153.9	20.1	62.2	43.3	3.86
**2019**	7	entry7052	8059717	75.2	117.0	98.0	175.0	17.9	48.6	49.4	3.85
**2019**	3	entry3056	8044987	77.4	116.8	99.0	159.9	21.7	50.9	48.2	3.85
**2020**	3	entry3054	7398245	70.9	108.0	107.3	344.6	21.2	66.8	52.4	5.33
**2020**	5	entry5031	8244791	73.0	108.4	97.8	322.9	16.8	51.7	50.3	5.16
**2020**	4	entry4046	8243776	68.0	107.3	99.3	424.5	16.5	62.1	45.4	5.16
**2020**	9	entry9025	8241671	70.0	108.4	98.0	354.5	16.3	42.8	44.6	5.11
**2020**	8	entry8031	8240862	71.6	112.3	92.5	346.9	18.5	57.0	49.5	5.10
**2020**	10	entry10025	8242637	72.3	114.7	96.5	363.2	17.2	53.4	54.4	5.10
**2020**	9	entry9011	8241417	69.0	113.3	95.6	372.5	17.2	42.0	52.0	5.08
**2020**	10	entry10002	304660	62.0	110.1	91.0	351.8	16.9	56.3	50.0	5.06

**2020**	7	entry7001	8247612	70.8	112.5	96.4	303.4	17.9	53.5	54.9	5.05
**2020**	7	entry7012	8239328	72.8	111.0	98.4	412.5	23.3	60.4	37.8	5.01
**2020**	9	entry9041	8241804	70.9	112.6	99.5	300.0	15.9	55.0	54.9	5.01
**2020**	9	entry9028	8241729	70.5	113.3	96.6	326.5	18.4	48.4	48.0	5.01
**2020**	9	entry9055	8242209	71.0	111.2	98.1	422.5	16.6	46.0	47.3	4.98
**2020**	7	entry7026	6175067	70.3	111.5	98.3	378.9	18.6	54.0	44.0	4.96
**2020**	7	entry7039	7400769	72.5	112.5	90.9	357.0	18.4	58.2	57.3	4.92
**2020**	4	entry4043	8243589	70.5	105.0	95.2	384.5	15.8	51.0	46.3	4.86
**2020**	8	entry8041	8241091	70.9	110.5	98.0	337.7	21.1	53.0	51.3	4.83
**2020**	8	entry8046	8241137	73.6	111.0	97.1	400.9	16.7	51.5	48.8	4.83
**2020**	9	entry9018	8241577	71.5	110.6	97.2	356.0	17.1	51.5	46.2	4.80
**2020**	3	entry3026	6175067	70.4	111.0	103.0	357.9	18.4	60.7	46.0	4.80
**2020**	10	entry10054	7398245	70.1	112.3	101.5	299.6	18.7	54.6	56.9	4.80
**2020**	10	entry10021	8242598	72.2	114.2	96.0	336.8	16.0	60.0	44.0	4.79
**2020**	9	entry9045	8241952	74.9	115.8	91.6	366.5	19.4	46.5	50.7	4.78
**2020**	9	entry9040	8241794	71.0	112.1	87.6	389.0	17.4	47.5	49.9	4.77
**2020**	8	entry8003	8240217	78.1	114.2	98.9	384.7	17.9	50.0	44.8	4.75
**2020**	9	entry9046	8241963	68.5	112.6	100.3	356.0	20.0	47.0	51.7	4.73
**2020**	9	entry9005	8241241	71.0	110.1	100.5	310.0	16.5	44.0	56.7	4.73
**2020**	8	entry8044	8241122	74.7	112.7	96.4	336.4	19.3	46.5	47.8	4.72
**2020**	8	entry8039	7400769	72.0	113.0	88.9	426.8	15.4	45.5	56.0	4.72
**2020**	9	entry9056	8242211	70.9	112.5	97.7	353.5	16.5	42.5	49.7	4.67
**2020**	4	entry4045	8243716	71.0	108.3	86.6	307.0	16.9	50.3	45.5	4.66
**2020**	11	entry11007	8048669	66.8	107.0	95.3	276.4	18.2	40.0	54.0	4.64
**2020**	9	entry9054	7398245	70.4	111.3	99.2	356.0	18.5	58.5	48.8	4.63
**2020**	9	entry9023	8241656	71.0	110.9	100.5	410.0	17.6	49.5	47.6	4.63
**2020**	9	entry9052	8242164	71.0	112.0	95.9	448.5	17.0	46.5	50.1	4.62
**2020**	6	entry6026	6175067	71.0	110.0	99.2	398.3	16.0	48.0	59.3	4.62
**2020**	10	entry10016	8242520	74.4	113.5	89.5	360.9	17.5	59.0	49.6	4.61
**2020**	8	entry8010	8240449	69.4	111.2	92.6	389.4	19.3	50.0	39.5	4.61
**2020**	10	entry10005	8242376	71.5	112.6	96.0	318.8	18.6	61.0	39.7	4.60
**2020**	7	entry7010	8239306	65.4	109.0	100.6	356.2	17.8	47.9	52.3	4.60
**2020**	8	entry8032	8240864	71.3	109.2	97.5	361.2	20.5	50.7	44.0	4.57
**2020**	11	entry11055	8054321	75.0	113.0	95.9	304.6	17.7	49.1	44.3	4.57
**2020**	4	entry4054	7398245	67.6	108.4	101.0	371.5	19.8	51.5	55.3	4.56
**2020**	8	entry8030	8240840	72.6	111.9	98.9	358.7	17.9	52.0	43.5	4.56

**2020**	7	entry7008	8239262	70.5	112.0	92.0	359.9	17.2	47.2	49.8	4.54
**2020**	8	entry8018	8240574	75.7	112.9	96.5	347.8	20.1	54.0	51.0	4.54
**2020**	6	entry6002	304660	62.8	106.5	92.1	363.3	17.5	46.6	47.7	4.53
**2020**	8	entry8040	8241055	71.9	111.5	95.1	430.0	18.7	54.5	46.3	4.52
**2020**	9	entry9010	8241408	72.0	113.4	95.2	294.5	20.0	60.5	45.0	4.52
**2020**	8	entry8025	8240790	71.4	107.2	99.5	384.9	15.4	52.0	44.3	4.51
**2020**	6	entry6030	8246525	70.7	110.0	100.3	416.2	16.2	46.0	40.8	4.51
**2020**	8	entry8036	8240873	68.4	109.6	93.1	376.6	15.8	54.3	50.0	4.51
**2020**	4	entry4037	8243467	71.0	110.4	98.7	424.5	18.9	45.6	40.2	4.50
**2020**	7	entry7005	8239230	72.0	112.0	89.2	378.5	16.1	52.5	64.1	4.50
**2020**	6	entry6039	7400769	72.5	114.5	97.3	420.6	18.5	48.7	45.5	4.49
**2020**	6	entry6005	8245477	70.8	110.5	95.1	359.8	18.5	46.5	39.2	4.49
**2020**	10	entry10009	8242393	73.7	112.0	91.0	332.1	16.7	58.0	48.9	4.48
**2020**	4	entry4044	8243684	81.5	115.6	92.6	370.0	19.0	56.4	42.0	4.48
**2020**	8	entry8007	8240287	72.5	113.3	97.4	344.0	18.7	43.7	41.3	4.47
**2020**	11	entry11023	8046597	70.5	110.0	88.7	376.6	18.3	49.1	50.3	4.46
